# Tuning of nanoparticle biological functionality through controlled surface chemistry and characterisation at the bioconjugated nanoparticle surface

**DOI:** 10.1038/srep17040

**Published:** 2015-12-01

**Authors:** Delyan R. Hristov, Louise Rocks, Philip M. Kelly, Steffi S. Thomas, Andrzej S. Pitek, Paolo Verderio, Eugene Mahon, Kenneth A. Dawson

**Affiliations:** 1 Centre for BioNano Interactions, School of Chemistry and Chemical Biology, University College Dublin, Belfield, Dublin 4, Ireland; 2 Department of Biotechnology and Bioscience, University of Milano – Bicocca, Piazza dela Scienza, 3. Milan 20126, Italy

## Abstract

We have used a silica – PEG based bionanoconjugate synthetic scheme to study the subtle connection between cell receptor specific recognition and architecture of surface functionalization chemistry. Extensive physicochemical characterization of the grafted architecture is capable of capturing significant levels of detail of both the linker and grafted organization, allowing for improved reproducibility and ultimately insight into biological functionality. Our data suggest that scaffold details, propagating PEG layer architecture effects, determine not only the rate of uptake of conjugated nanoparticles into cells but also, more significantly, the specificity of pathways via which uptake occurs.

The reproducible synthesis of nanoparticles using oligomeric moieties that limit non – specific association with biological fluids, and which also facilitate the grafting of antibodies or other biomolecules at the nanoparticle surface, is seen as a basic requirement for the control of biological interactions^1^,^2^,^3^,^4^. Still, it remains a challenging task to precisely reproduce batches of nanoparticles across independent syntheses. In addition, the degree to which relatively subtle surface structural variations can affect interactions at the cell and barrier levels leading to, for example, substantially different cellular uptake and receptor specificity is poorly understood. Here we have used a silica – polyethylene glycol (silica –PEG) based bionanoconjugate synthetic scheme to study the subtle connection between receptor specific recognition and architecture of surface grafted layer. The level of reproducibility and characterization allows us to suggest that the architecture of the PEG layer determines not only the rate of uptake of conjugated nanoparticles into cells but also, more significantly, the specificity of pathways through which uptake proceeds. This in turn suggests the need to develop more refined methods of nanoparticle surface grafting and characterisation when the application involves biological targeting.

## Results

Internally fluorescently labelled silica particles were prepared and surface modified using different concentrations of amino alkoxysilane molecules in aqueous conditions. Succinimidyl-([N-maleimidoproprionamido]-octylethyleneglycol)ester (SM(PEG)_8_), a heterobifunctional linker, was than reacted with the amine presenting surfaces, producing a PEGylated surface displaying terminal maleimide groups, subsequently reacted with a thiol grafted protein (we use the protein Transferrin in this example). As we now outline, exploration of the parameter space, for this general approach, allows us to tune surface structure, which is then shown to play a definitive role in the ultimate biological behaviour.

**Figure 1:**
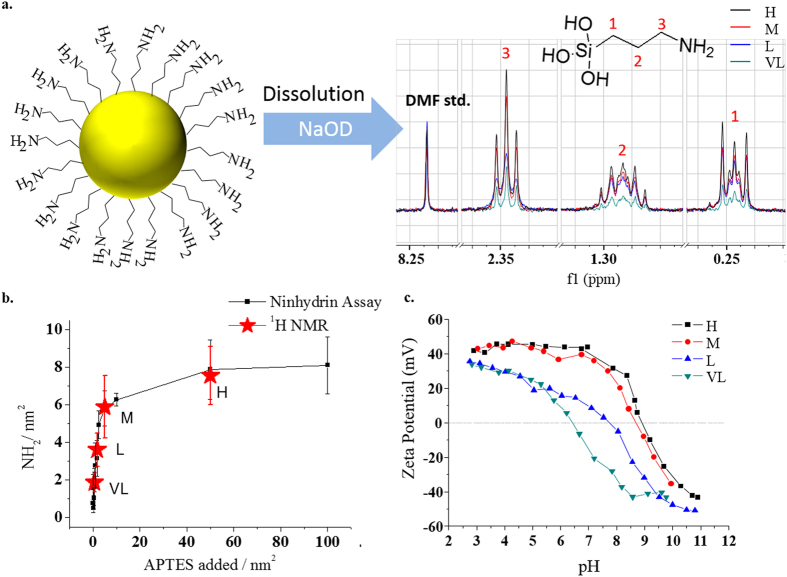
*Amination control* (**a**). Representative NP dissolution NMR corresponding to chosen amine surface densities labelled H, M, L and VL (**b**). Ninhydrin assay measurement of no. of surface amines across precursor concentration range in optimal amination conditions (“natural” pH with thermal treatment). Red stars indicate the chosen surface densities with corresponding NMR surface measurements (All Ninhydrin and NMR averaged from minimum of three independently synthesized batches) (**c**). Representative zeta potential–pH titrations for chosen surface densities.

Aminopropyltrimethoxysilane (APTS) was surface condensed from well – mixed aqueous mixtures^5^ (see Fig. S1) varying the reaction concentration used to control the final amine surface density. Multiple batches of amino functionalized nanoparticles were thus synthesized and studied using a modified ninhydrin assay^6^ and zeta potential/pH titrations (see Fig. 1b,c, also methods section S3). Some such assays, in our studies of nanoparticle surfaces, proved variable and irreproducible, depending on the specific method chosen, and we caution against their use, unchecked, in general. While we have been able to adapt the Ninhydrin assay (see also Fig. S2) to obtain variations of less than 10% assay to assay, an independent quantitative measure of ligand density remains necessary. Using solution proton nuclear magnetic resonance (NMR) on the intact nanoparticles monodispersed in D_2_O, at sufficient NP concentration (1% w/w for 50 nm), one can detect the relaxation of the propyl chain (C-H) protons of bound APTS (Fig. 1), but the signals are low and broadened. Silica solubility is known to rapidly increase above pH 11^7^,^8^. Accordingly, addition of aliquots of sodium deuteroxide solution (NaOD) to the aforementioned samples resulted in the solubilisation of the silica nanoparticle cores (Fig. S3 and S4), and release of the surface ligands. The resultant increase and narrowing in the corresponding NMR signals permitted peak integration of the ligand protons and, referencing to a calibration curve (normalized by internal standard) (Fig. S5) for fixed acquisition times, a direct measure of the amine surface density. The results were found to be in good agreement with (modified) Ninhydrin studies (Fig. 1b). Four representative amine densities, nominally high, medium, low and very low (**H**, **M**, **L** and **VL)** corresponding to mean densities of 8, 5.9, 2.5 and 0.8 NH_2_ groups per nm^2^ (measured by ninhydrin assay and averaged from 9 individual batches), were chosen as representative candidates for further investigation. Reproducibility was emphasized through the careful control of synthetic parameters and was monitored through consistent investigation of batch –to – batch variations (Fig. S6 for sample batch results), which were found to be quite modest, while efficiency of purification was also monitored by NMR (Fig. S7). In our experience, these silica scaffold particles are amongst the most reproducibly synthesized and characterized of their type, and their study, across multiple batches, reveals subtle roles of surface architecture in biological interactions.

**Figure 2:**
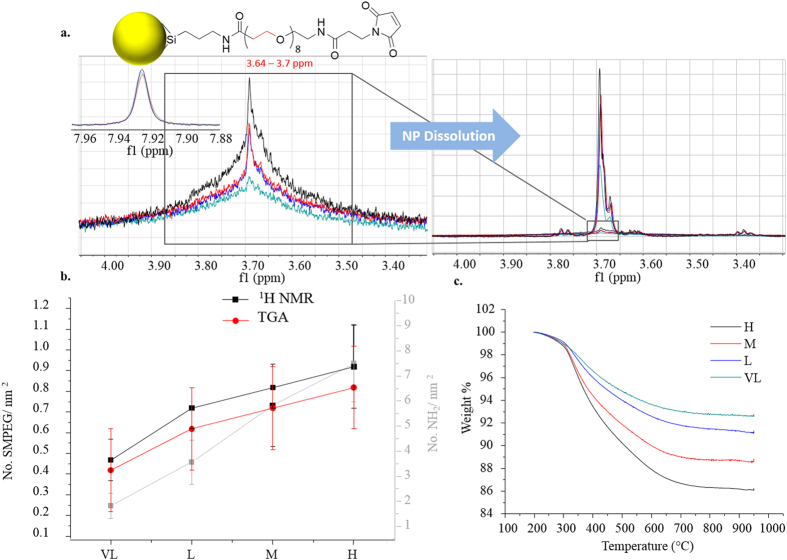
*Functionality control by varying surface amine density* (**a**). NMR of PEGylated H, M, L and VL nanoparticles before and after dissolution with all intensities normalized against an internal standard. (**b**). Comparison of SM(PEG)_8_ determination by NMR and TGA with amine density in background (grey) for series H, M, L, VL. (**c**). Representative TGA data for NP set. (**d**). Table 1.

In the same way, NMR (Fig. S8 for assigned spectra) was then applied in the determination of surface ligand concentrations for subsequent conjugation steps, and proved the most powerful of the range of complimentary methods used in the study of SM(PEG)_8_ coupling to amine presenting NPs. For the aminated nanoparticles (**H**, **M**, **L** and **VL**), reactive SM(PEG)_8_ linker concentrations were varied, with products then purified by centrifugation (Fig. S9). Ligand surface densities were investigated indirectly by Ninhydrin assay (monitoring residual reactive amine) and directly by dissolution NMR (Fig. S10) and thermogravimetric analysis (TGA). Thus, PEGylation was quantified by proton NMR (Fig. 2a,b, Table 1 and Fig. S9 for details) using the ethylene protons from 3.64 – 3.7 ppm after NP dissolution. TGA was used as a complimentary method (Fig. S11) which allowed for the determination of relative amounts of the ligands with results in fair agreement with those of NMR (see Table 1). The Ninhydrin assay, applied post – PEGylation, appears to under – detect unreacted amine groups (when compared to NMR) which we attribute to limitations in surface accessibility of the Ninhydrin reagent to the PEGylated surface (Fig. S12). PEG surface coverage was interpreted though dispersion stability studies using dynamic light scattering (DLS), differential centrifugal sedimentation (DCS) and serum binding resistance investigations (SDS – PAGE). In all cases the starting amine nanoparticles were highly unstable, agglomerating immediately in phosphate buffered saline (PBS). We studied varying surface PEG concentration equivalents against each amine density and found that higher levels of PEGylation, as could be expected, result in an increase in stability against agglomeration in PBS (Figs S13 and S14 for transmission electron microscopy – TEM). Additionally protein adsorption studies, using serum incubation, were used as a probe for surface coverage and showed, as expected, a reduction in protein adsorption levels for increased SM(PEG)_8_ densities (see Fig. S12d)^9^,^10^,^11^.

It was apparent that increased SM(PEG)_8_ surface density derived from increased surface amine density (Fig. 2b grey line), although in a non – linear way (see Table 1). For the highest amine densities ~13% NH_2_ could be maximally occupied post – conjugation, while for lower densities ~50% were, indicating a surface steric crowding as a limiting factor when approaching maximal coverage.

**Figure 3:**
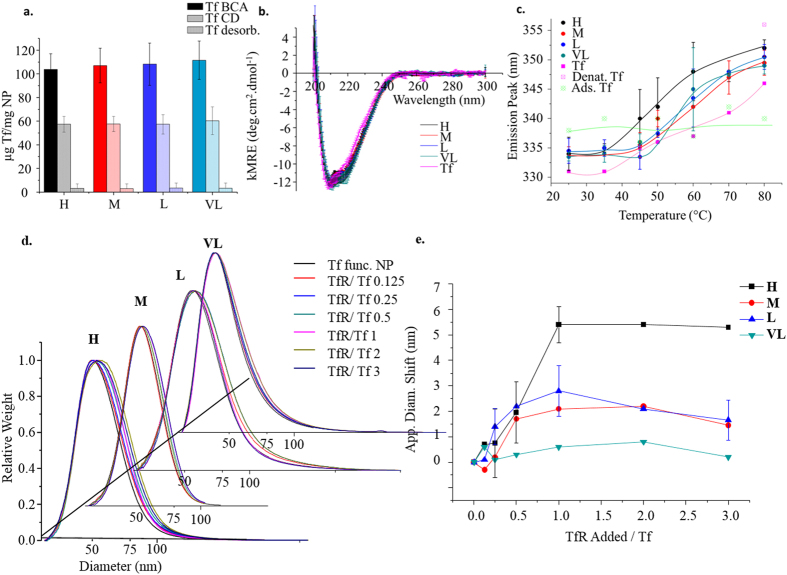
*Bioconjugate Characterization* (a). Protein concentration determined by BCA assay and Circular Dichroism (**b**). Overlapped curves for concentration normalized Circular Dichroism (H, M, L, VL and Tf), averaged over five separate batches for each of H, M, L and VL (mean ± std. dev). (**c**). Temperature denaturation curves plotting tryptophan emission maximum against temperature. (**d**). Representative full DCS dispersion size distribution shifts with increasing concentration of soluble TfR for nanoparticles (H,M,L and VL) with a shift in the particle size obtained by incubation (1 h at 37 °C) with TfR at varied concentration (see methods) (**e**). corresponding averaged binding curves (error bars indicate points averaged from a minimum of three experiments).

We next coupled the biological moiety (Tf) to the terminal maleimide groups in conditions of ten times protein monolayer excess. We determined the efficiency of centrifugal washing procedures (Fig. S15) and the concentration of surface bound Tf by colorimetric protein assay (microBCA) and Circular Dichroism (CD) (Fig. 3a, S15 and S17). Interestingly, we noted across all methods (further supported by the fluorescence intensities in our tryptophan denaturation studies) that Tf coupled at a saturating concentration almost independent of PEG surface density (90–110 μg/mg NP by microBCA assay). This observation can be explained by the availability of a large excess of reactive maleimide functions at the surface, with the limiting factor being steric crowding of the protein itself.

Initially, numerous sets of nanoparticle conjugates (>300) were studied for biological behaviour by comparing total uptake in adenocarcinomic human alveolar basal epithelial cells (A549) and, more specifically, the percentage internalised via Tf receptor mediated processes^1^ (see Fig. S16). As we show later this study revealed that modest variations in surface structure are manifested in distinctions in overall cellular uptake, and furthermore, that there may be more biologically subtle surface effects than have previously been noted^3^.

Aiming to elucidate the relationship between functional surface architecture and biological behaviour, we identified a set of optimal conditions that were illustrative of the effects. We applied extensive measurements across multiple batches of the four amine density variations (**H**, **M**, **L** and **VL**) reacted with excess SM(PEG)_8_/ NH_2_ stoichiometric ratio of 1.9/1 (as highlighted also in Fig. S16).

We examined features of protein ligand structural stability by CD (see Fig. 3b and S17) and Tryptophan fluorescence denaturation studies figure (Fig. 3c and S18), noting no substantial differences which could be clearly correlated to *in vitro* biology. Furthermore, we tested colloidal stability (see Fig. S19) and nanoparticle structural stability (see Fig. S20), applying them, where feasible, to the time – course of typical biological experiments. In these experiments, investigating particle and conjugate protein structurally, no decisive differences were noted between different particle types.

By Differential Centrifugal Sedimentation titration against soluble transferrin receptor^1^ (sTfR), we noted reproducible binding behaviours across independent batches (see Fig. 3d,e and S21). While surface bound Tf remained consistent between batch and type, it is apparent that the highest receptor binding function occurs in the case of **H,** corresponding to the highest amine density and consequent highest SM(PEG)_8_ density (see Table 1).

**Figure 4:**
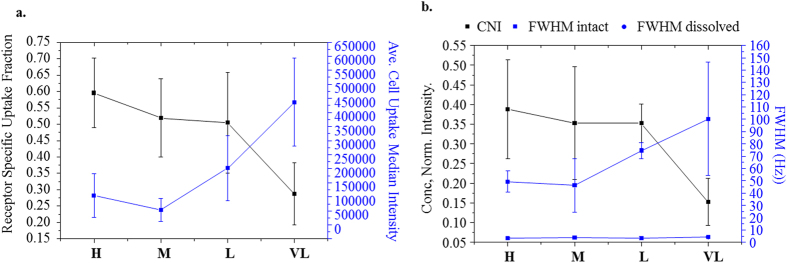
*Cell Biology correlates to PEG layer NMR characteristics*. (**a**). Specific uptake Fraction (mRNA silenced component/total uptake component) for particles with different amine densities (H, M, L and VL) (each point average of more than 5 independent batches) (black line) and average median intensities associated with cellular uptake (blue line). (**b**). NMR signal parameters for particle series (H, M, L and VL), showing ligand concentration normalized integration (CNI), FWHM intact and FWHM following nanoparticle dissolution.

This binding behaviour mirrors *in vitro* cell uptake experiments (referred to as “receptor specific frac.” in Fig. 4a). The receptor specific endocytosis of particles was measured by comparing particle uptake into cells with reduced expression of TfR with the uptake into a control population (see supporting section “*Cell silencing and flow cytometry”* for full details).

Deducing that this trend in biofunctionality is unrelated to the amount of functional units (Tf) attached (see Fig. 3a), nor dramatic structural imposition upon conjugation (Fig. 3b,c) it is likely linked to the structural details of the underlying conjugating PEG layer.

We gained further insight here through our NMR studies (Fig. 4b). By Lorentz fitting we measure the Full – Width Half – Maximum (FWHM) of the PEG backbone peak (3.5 – 3.9 ppm) (Fig. 4b, blue line) and compare it to the concentration normalized integration (Fig. 4b black line). It should be noted that although ligand signal broadening due to nanoparticle immobilization has been previously observed^12^,^13^, here the precise interpretation of the broadening, and linkage to relaxation effects at the nanoparticle surface is incompletely understood. However, it is believed that the changes in magnitude and shape of the signal for ligands when attached at particle surfaces can be related, at least in part, to increases in the spin – spin relaxation component (T2, dephasing relaxation) and wider distribution in chemical shifts due to differences in ligand binding sites environments on the nanoparticle surface^14^. As a rough guide, typically the FWHM is 3 Hz for dissolved nanoparticles versus >50 Hz for intact nanoparticles, so the effects are highly significant (Figs 2a and 4b). The degree of broadening decreases and integrated signal intensity, normalized by concentration of ligand, increases as the PEG density is increased from **VL** to **H**. This suggests increased homogeneity in the ligand environment and decreased T2 type relaxation for higher PEG densities. Supported by Tf binding studies (Fig. S22) the lower density PEGylated surfaces maybe more accessible to ligand – surface association. However no fully microscopic picture is possible based on these results alone.

## Discussion

Nanoparticle surface interactions have previously been reported to greatly influence protein structure and hence function^2^,^3^,^15^,^16^,^17^. However CD results (see Fig. 3b), though by no means a sensitive tool for biological function, may show minimal impact on secondary structure as for example **VL** to **H**. Tryptophan fluorescence denaturation studies^18^ (see Fig. 3c and s18) also depict quite similar denaturation behaviour across the series, although **H** (black line) shows some propensity for denaturation at a lower temperature than other types, where surface interactions are known to retard denaturation of proteins (compare surface adsorbed Tf (Ads. Tf)).

Despite these observations there is no compelling measurable that would suggest significant biological differences observed in the cell studies presented in Fig. 4a above. Before discussing these results in further detail, we summarize briefly some background. Broadly speaking we understand nanoparticles when functionalised by biomolecules to enter biological cells by a variety of uptake pathways, in distinction to the biomolecules which typically enter via only a single pathway^19^,^20^. The origin of such differing pathways for nanoparticle entry is likely due to the heterogeneity of the presentation and organization of the surface ligands, the size of the particles, and many other factors, as yet undetermined. In a typical experiment using RNA interference, where the gene silencing is highly effective (see reference 1 and associated supplementary), we can estimate the role of the target pathway in the uptake of nanoparticles by comparing otherwise identical cells with and without the presence of (as in this case) the receptor. Thus we can obtain the fraction of uptake that is specific to the targeted receptor. There are of course many controls and details associated with such experiments, which in this particular pathway have been previously worked out and reported in detail^1^.

In Fig. 4a, the blue curve, indexed from the right, shows how the amount of cell uptake varies with underlying PEG grafting density, for fixed surface grafted protein (Tf) concentrations. The black curve indexed from the left illustrates the fraction of uptake mediated by the transferrin receptor. It is important to distinguish between the two measurables (as described above). Many non – specific effects can be shown to affect the total amount of uptake, including even the nature and amount of serum added^1^,^21^. The specificity ratio is a more delicate assessment of the degree to which nanoparticle cell interactions are able to promote uptake via a targeted pathway. Here, we observe the evolution of that ratio with surface PEG density and organization (see Fig. 4a,b, black lines). It is particularly striking to note the degree to which the surface characteristics, determined by NMR of the PEG layer, are capable of parameterizing the receptor specific uptake. This distinguishes the above measurement from all other physicochemical measurements presented prior, and many typical ones that are not stressed here. Thus, to summarize, the line broadening characteristics parameterizes the actual amount of cell uptake. Much more strikingly, if we normalize the signal intensity for the immobilized layer with the actual PEG surface density, we find a curve that parameterizes precisely the fidelity of the uptake though the transferrin pathway.

We do not wish to over interpret these results and caution against doing so, for it is not yet possible to relate with any certainty, the NMR signal characteristics (relaxation times) to the underlying nanoparticle surface local microscopic structure. However, it is of some interest to note that surface layer relaxation times could be a sensitive determinant of nanoparticle – receptor interactions, providing a new window into the potential to characterize nanoparticle targeting surfaces. Perhaps more generally, and more broadly significant, we have observed here that whatever the apparent advantages of modifying the targeting ligand density to achieve greater cellular uptake, the issue of how precisely that uptake is targeted to a specific pathway is a different question, and is based on quite different design features. It is of little doubt that those factors will greatly affect the off – target interactions when nanoparticles are presented to many different cells, or tissues. We consider that in future when designing targeting constructs, more attention might be paid to the fidelity and specificity of uptake and especially those physicochemical methods that might be sensitive to those features.

## Methods

### Silica nanoparticle synthesis

Silica particles were synthesised using the Stöber method. To 25 ml of ethanol (99.9%) 0.91 g of NH_4_OH (28.0–30.0% NH_3_ basis) was added in a polypropylene container. To this mixture, under rapid stirring, was added 500 μl of the prepared FITC-APTMS conjugate solution. The reaction was stirred for 15 minutes, upon which Tetraethyl orthosilicate (TEOS) (940 μl) was added. The reaction was then stirred at 600 rpm at 25 °C for a further 20 hours in darkness. The resulting nanoparticle suspension was centrifuged down at 14,000 rpm for 20 minutes, with the pellet then resuspended in fresh ethanol aided by bath sonication. This washing procedure was repeated twice more, followed by 3 water washes and a final resuspension in water at a total volume of 12 ml.

### Surface Amination

The silica particles synthesised and washed were dispersed at a concentration of 10 mg/mL in Milli-Q water following which the appropriate excess amounts of APTES were added. The dispersion was left to shake at 25 °C for 1 hour after which the temperature was increased to 90 °C. The dispersion was washed with Milli-Q water for four centrifugation cycles. Further details for the procedure can be found in SI.

### Protein grafting onto nanoparticles

#### Step 1: PEGylation of NH_2_ functionalized silica nanoparticles

Amino functionalized nanoparticles were washed twice with HEPES buffer (20 mM pH 7.4) with redispersion in the same buffer at a final concentration was 10 mg/mL. Succinimidyl-([*N* maleimidoproprionamido]-octylethyleneglycol)ester (SM(PEG)_8_) (Thermo Scientific) was diluted in HEPES prior to mixing with the nanoparticle dispersion. The SM(PEG)_8_ solution was added to the particles at a 1 to 1 volumetric ratio giving a final particle reaction concentration of 5 mg/ mL. The mixture was left shaking at 700 rpm and 25 °C for 2 hours after which it was washed with HEPES buffer (20 mM pH 7.4). Full details about buffer preparation and Step 1 can be found in SI.

### Step 2: Modification of protein (Transferrin) with SATPEG_4_

Holo – Transferrin was dispersed in PBS pH 7.4 (5 mg/ mL, 62.5 μM) and to it N-Succinimidyl S-acetyl(thiotetraethylene glycol) SAT(PEG)4 (Thermo Scientific) was added in a 1:1 molar ratio. The solution was left slowly shaking at 400 rpm, at 25 °C for 30 min after which time a deacetylation solution was added (0.1 mL/mL reaction) and was left to react for a further 2 hours.

Following this modification purification was performed using pre-packed NAP 25 Sephadex (GE Healthcare) columns which were pre equilibrated with HEPES 7.4 for three column volumes. Tris(2-carboxyethyl)phosphine (TCEP) (1:1 TCEP to Transferrin molar ratio) was added to the protein after purification and the solution was left for 5 minutes. Modified protein was combined with NP-PEG-Maleimide immediately following this time period. For full details on buffer preparation and protein modification see SI.

### Step 3: Nanoparticle – Protein conjugation

The nanoparticle dispersion was added to the modified protein solution to give a final mass ratio of 1:1. The dispersion mixture was then left for 2 hours at 400 rpm shaking at room temperature. 2 – mercaptoethanol was added to the dispersion (1 mM final concentration) which was then left for 5 minutes. Finally the particles were washed four times with PBS buffer and stores at 4 °C. The full details on step 3 can be found in SI.

### Receptor binding studies

To increase the colloidal stability in PBS across batches Bovine Serum Albumin (BSA) was added to all samples at a final concentration of 0.1% (wt/wt).

Lyophilised transferrin receptor aliquots were by adding 250 μL of filtered PBS 7.4. In a typical titration, 20 μL Tf grafted nanoparticles (10 mg/mL) were added to 10 μL of 1%(wt/wt) BSA and left for 5 mins to equilibrate After which they were diluted to a final volume of 100 μL with either PBS or a PBS TfR mixture. The final dispersion consisted of particles, BSA and TfR was 2 mg/mL, 0.1%, and 1 – 60 μg/mL respectively. The particles were incubated with receptor for 1 hour at room temperature before analysis by differential centrifugal sedimentation (DCS). Full details about the incubation and DCS procedure can be found in the SI.

### Cell culture

The adherent tumour cell line A549 were maintained in monolayer cultures in MEM supplemented with 10% fetal bovine serum. Cells were cultured in an incubator at 37 °C with 5% CO_2_/95% air and saturated humidity. Cell line was confirmed to be mycoplasma negative using the MycoAlert™ Kit (Lonza) and were tested monthly.

### Cell silencing and flow cytometry

A total of 13,000 cells were seeded in 24 well plates and incubated for 24 hours prior to silencing of the gene coding for transferrin receptor (TFRC). Cells were transfected with 15 pmol of Silencer Select siRNA siTFRC using Oligofectamine™ according to the manufacturer’s instructions. Neg1 silencer was used as a negative control. Cells were transfected with siRNA for 72 hours prior to exposure to nanoparticles or labelled transferrin.

To exposure the cells to nanoparticles, after 72 hours silencing, cells were washed for 20 minutes in serum-free MEM. The medium was aspirated and nanoparticle suspensions were applied to the cells. Nanoparticle suspensions were freshly prepared by diluting the nanoparticle stock solution in serum-free MEM. Similar experiments were performed by exposing cells to a solution of 5 μg/ mL Alexa488 labelled human transferrin in serum-free MEM.

For flow cytometry, the cells were washed once in MEM supplemented with 10% FBS (v/v), twice with PBS and harvested with trypsin. Cell pellets were fixed at room temperature with 4% formalin (Sigma-Aldrich) in PBS for 20 minutes and resuspended in PBS. Cell-associated fluorescence (15,000 cell per sample) was detected using an Accuri C6 (BD Biosciences). The results are reported at the median of the distribution of cell fluorescence (excitation: 488 nm, filter: 530/30 nm), averaged over 2–3 independent replicates. Error bars represent 1 standard deviation of the mean between replicates.

The receptor specific fraction is determined by the difference in median cell fluorescence intensity (following 6 hours exposure) between the non-silenced control and silenced cells, divided by the non-silenced control intensity as reflected in Eq 4 in SI. For more details see SI.

## Additional Information

**How to cite this article**: Hristov, D. R. *et al.* Tuning of nanoparticle biological functionality through controlled surface chemistry and characterisation at the bioconjugated nanoparticle surface. *Sci. Rep.* **5**, 17040; doi: 10.1038/srep17040 (2015).

## Supplementary Material

Supplementary Information

## Figures and Tables

**Table 1:** 

	Ninhydrin	NMR		TGA
	NH_2_/nm^2^	NH_2_/nm^2^	PEG/nm^2^	PEG/nm^2^
**H**	8.0 ± 1.6	7.6 ± 1.3	1.0 ± 0.1	0.8 ± 0.2
**M**	5.9 ± 1.5	6.1 ± 1.3	0.9 ± 0.2	0.7 ± 0.2
**L**	2.5 ± 1.0	3.7 ± 0.7	0.7 ± 0.2	0.6 ± 0.2
**VL**	0.8 ± 0.4	1.8 ± 0.6	0.45 ± 0.2	0.4 ± 0.2
