# Supplementary material for: Tuning of nanoparticle biological functionality through controlled surface chemistry and characterisation at the bioconjugated nanoparticle surface
Source: Sci Rep. 2015 Dec 1;5:17040. doi: 10.1038/srep17040 (PMC4664868; doi:10.1038/srep17040)
Supplement: Supplementary Information [file srep17040-s1.pdf]

Supplementary Data

Tuning of nanoparticle biological functionality through controlled surface chemistry and characterisation at the bioconjugated nanoparticle surface.

Delyan R. Hristov, Louise Rocks, Philip M. Kelly, Steffi S. Thomas, Andrzej S. Pitek, Paolo Verderio, Eugene Mahon* and Kenneth A. Dawson*.

Centre for BioNano Interactions, School of Chemistry and Chemical Biology, University College Dublin, Belfield, Dublin 4, Ireland

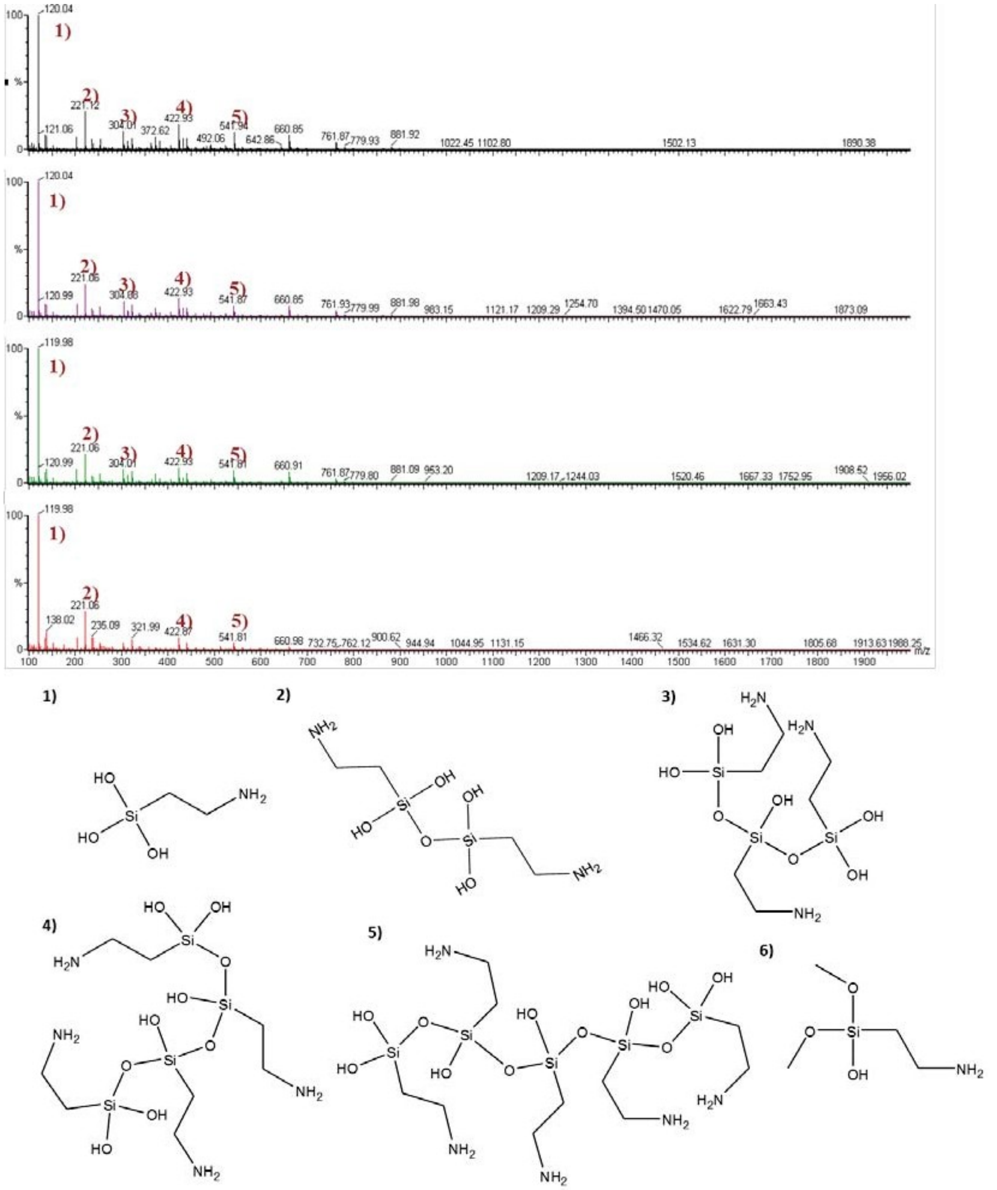

Figure S1. ESI-MS of APTS 1% wt/wt in water for 120 minutes (unbuffered, pH 9.5) at times 5, 50, 90 and 120 minutes (top to bottom) with beneath suggested assignments.

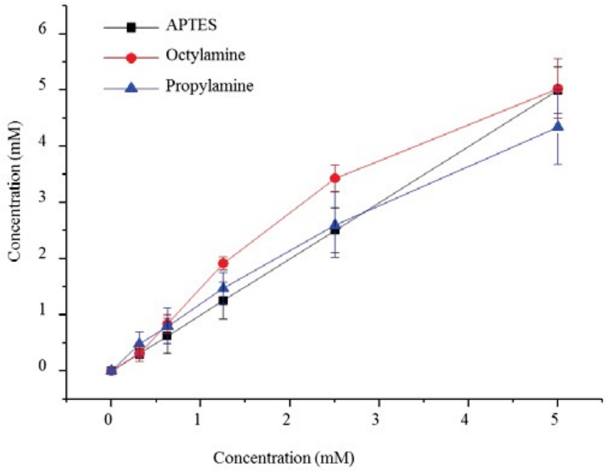

Figure S2. Ninhydrin standard curve validation for amine concentration determination

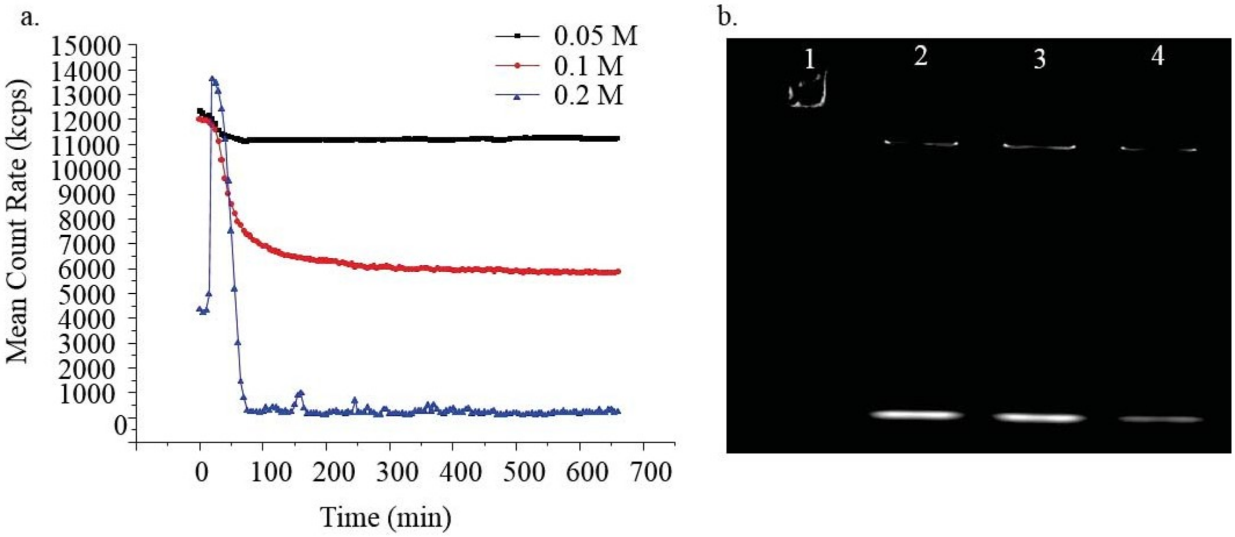

Figure S3. NP dissolution experiments for varied NaOH concentrations a. Monitoring over time by DLS (Malvern ZS) mesauring mean scattering count rate at fixed signal attenuation (final “dissolved” count rate of around 100 kcps where DI water gives 30-50 kcps). b. Indication of core FITC-silica NP dissolution by dye release. Lane 1 time 0 (no NaOH), lane 2,3,4 are 3, 6 and 24 hours incubation (1% silica in 0.2M NaOH at 37°C) respectively.

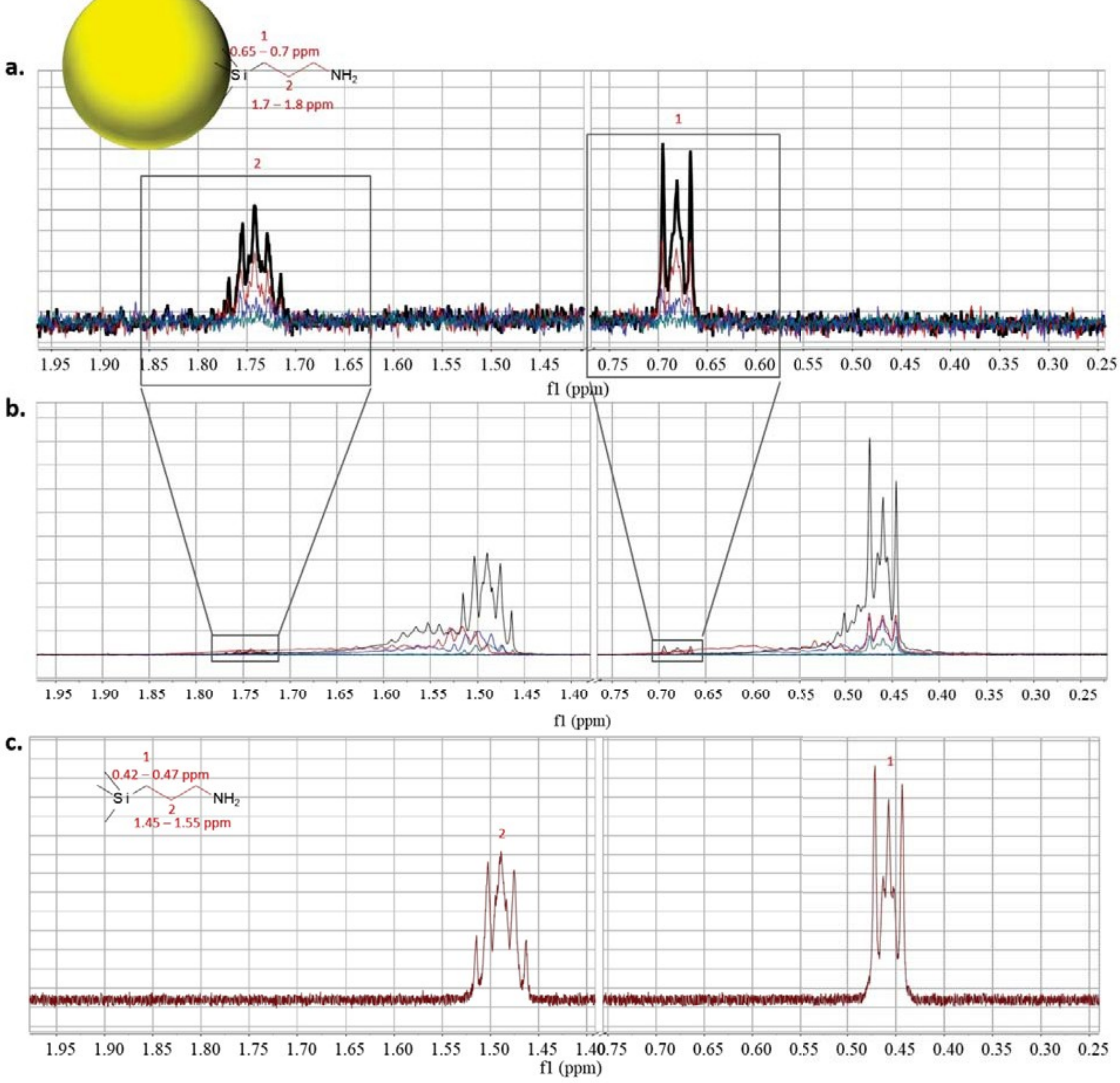

Figure S4. ^{1}H NMR studies on amine functionalised silica nanoparticles. a. NMR on intact NPs gives signals at 0.65-0.7 ppm and 1.75-1.8 ppm corresponding to propyl chain C*H*2 (1 and 2 counting from Si). b. Dissolution results in a large increase in signal intensity and change in chemical shift by about 0.2 ppm due to solubilisation of surface ligands. c. corresponding control signals for APTS alone

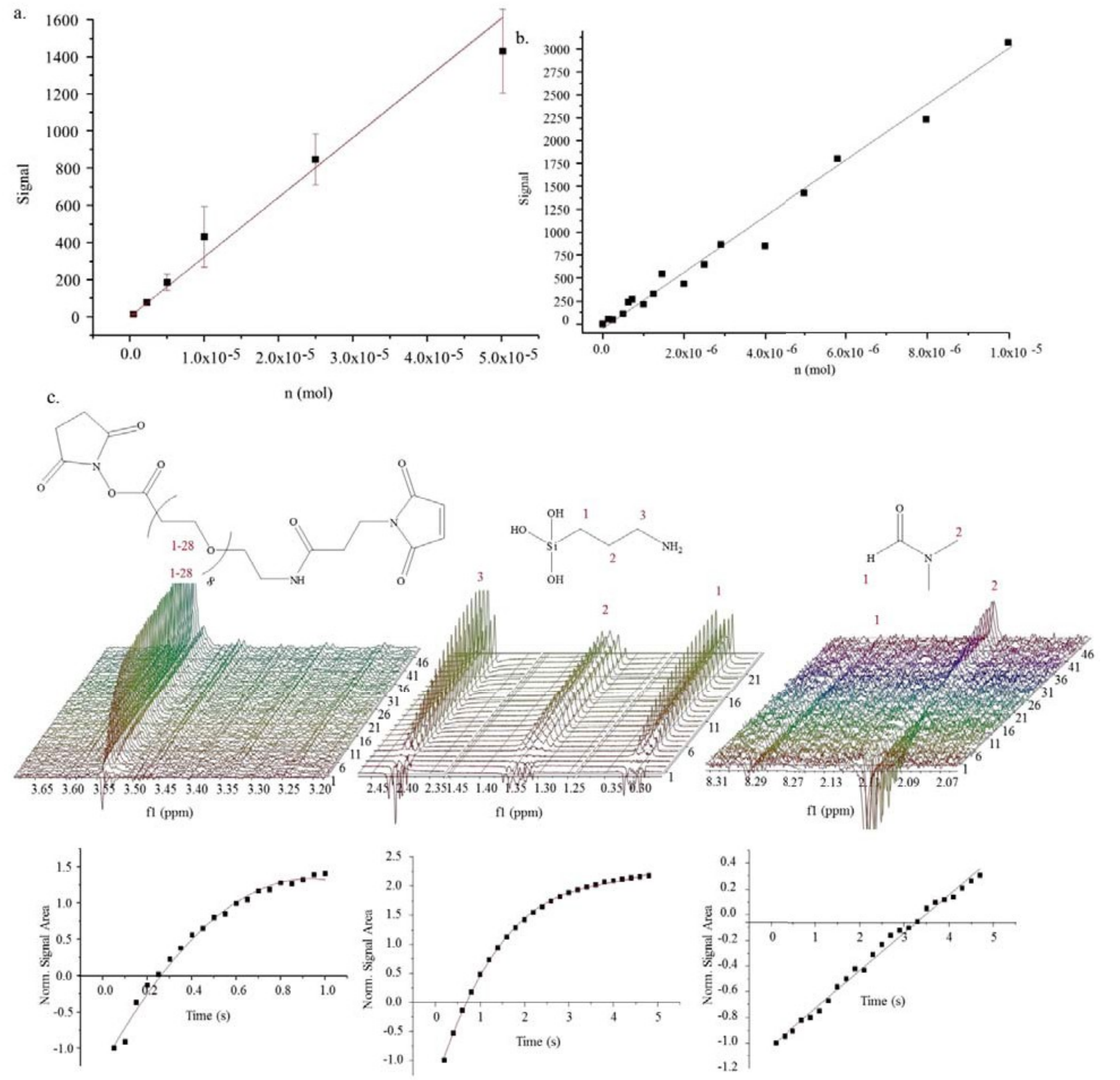

Figure S5. Calibration line and equation obtained from known concentrations of a. APTS and b. SMPEG8. c. T1 relaxation time analysis by inversion-recovery method was used to ensure validity of concentration – signal dependance. T1 relaxation times (where T1= approx. 1.44[illegible]$_{nul}$) obtained are 0.3s, 0.8s and 4.2s for SMPEG8, APTS and DMF respectively. Delay times between T1 measurements were 0.05s, 0.2s and 0.1s seconds left to right respectively. 10 second acquisition time was then used for quantifying concentrations from ^{1}H NMR

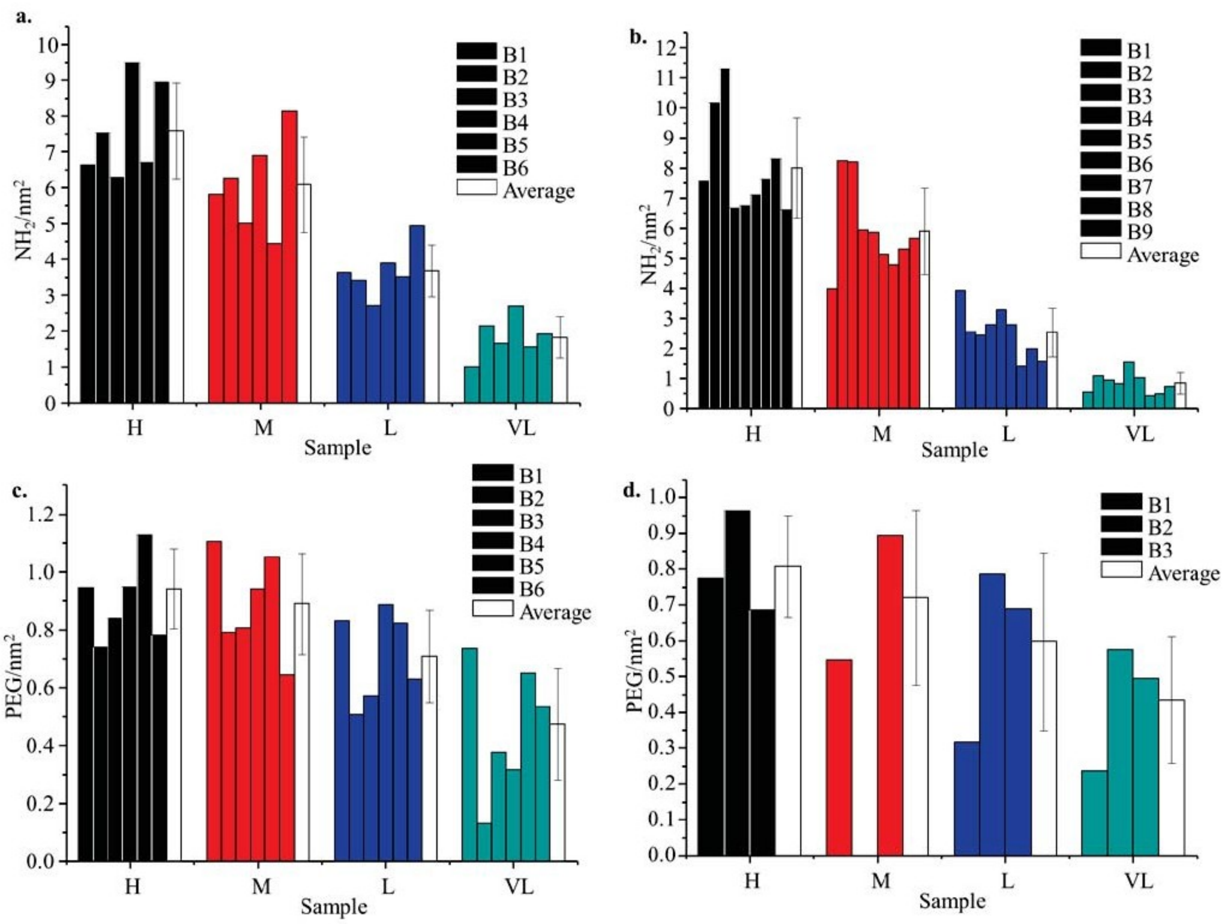

Figure S6. Batch to batch reproducibility determining amine density by a. NMR and b. ninhydrin assay and SMPEG density by c. NMR and d. TGA.

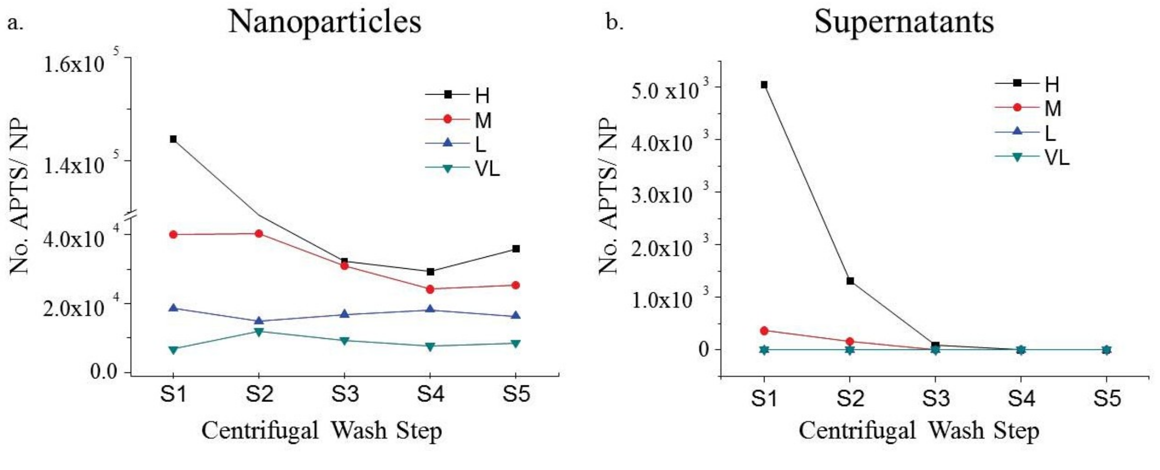

Figure S7. Validation of washing steps following amine functionalization by ^{1}H NMR a. NP fraction immobilised surface amine concentration following each washing step. b. Residual supernatant APTS concentration following each step.

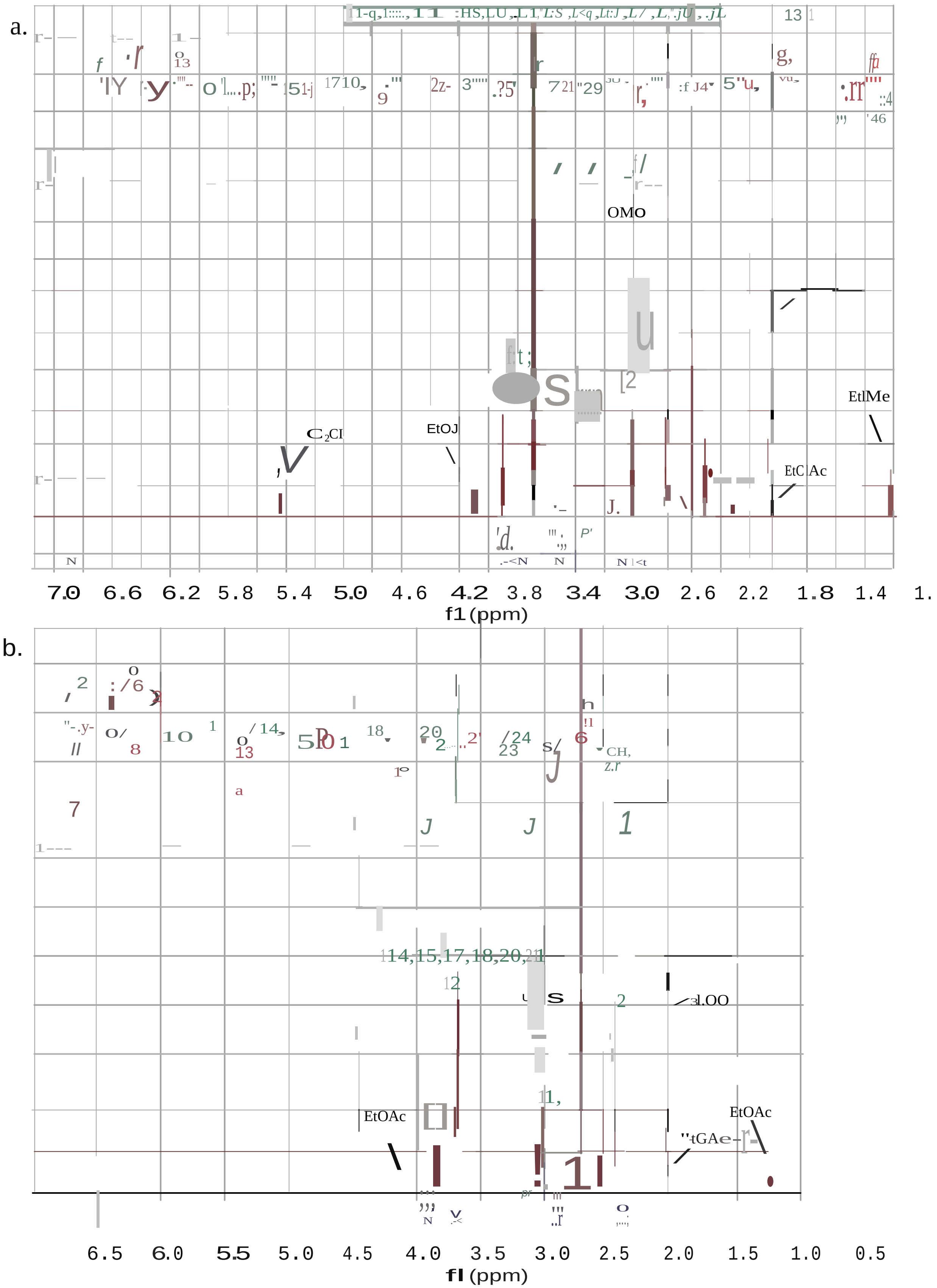

Figure SS. Assigned NMR spectra of PEG linkers employed in functionalisation a. SMPEGS and b. SATPEG4

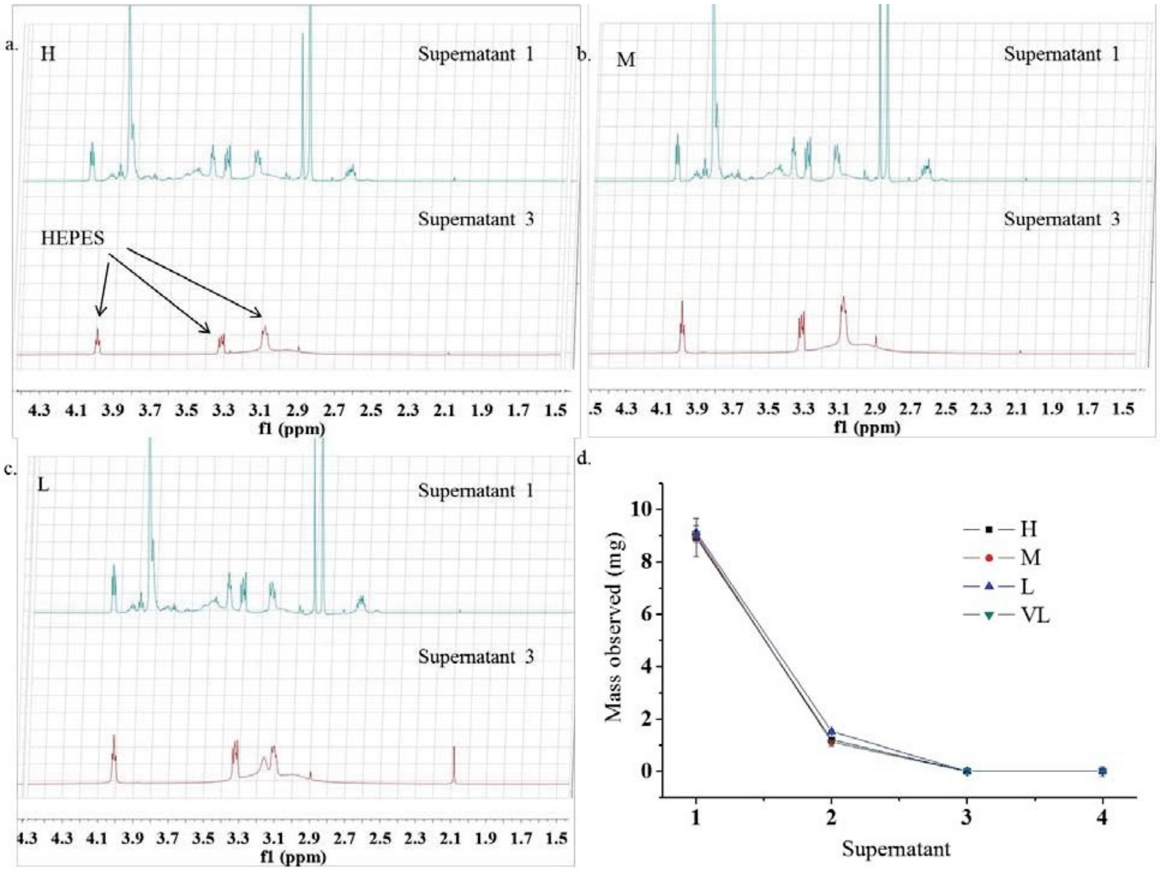

Figure S9. Following surface reaction, excess PEG removal monitored by NMR and microbalance. NMR of the first supernatant (supernatant 1) and the third supernatant (supernatant 3) of the washes of representative H a., M b. and L c. particle batches compared with HEPES controls and d. mass in supernatant following drying measured by analytical microbalance (sartorious microbalance).

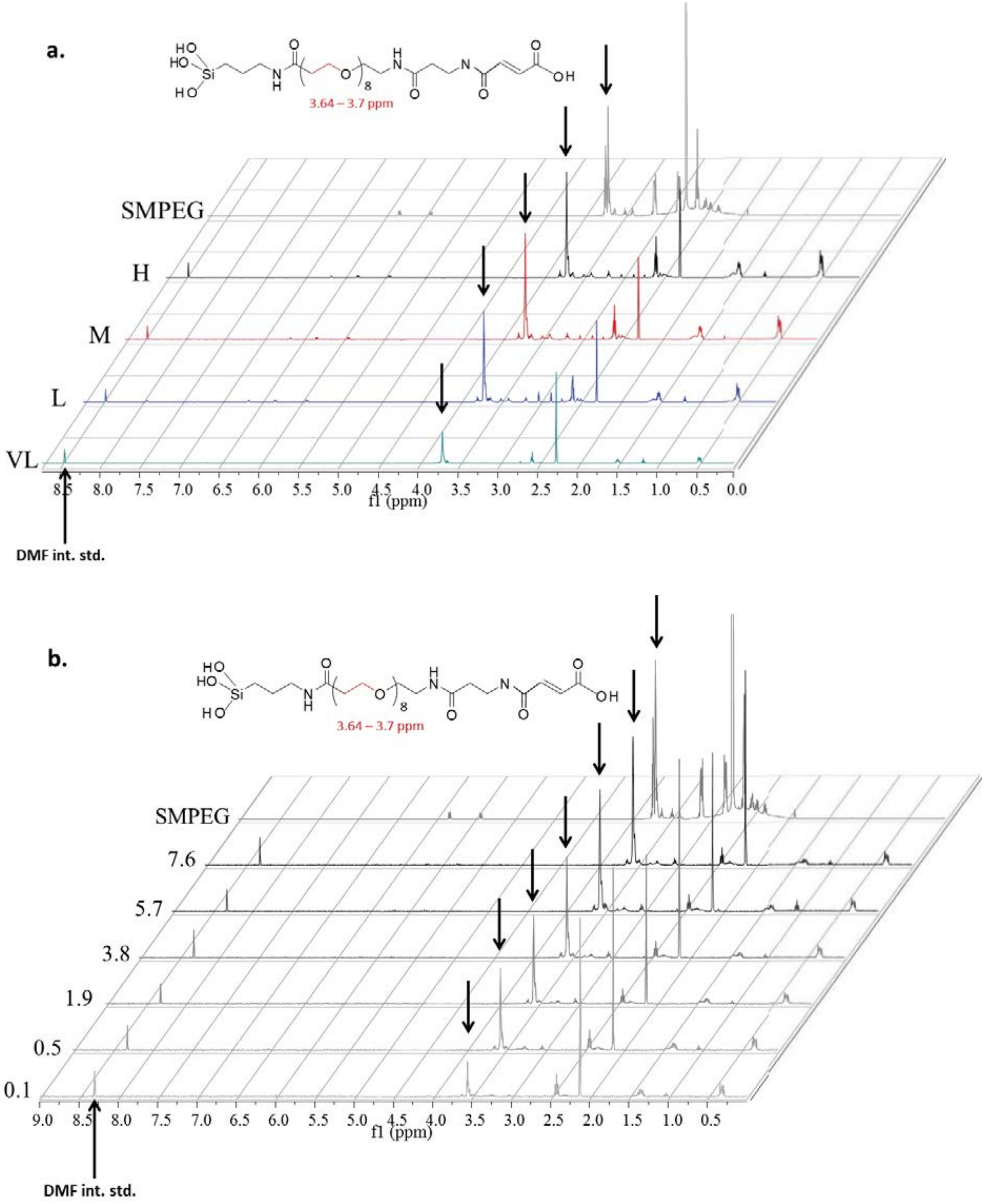

Figure S10. Full dissolution ^{1}H NMR Spectra stack for a. HMLVL dissolved PEGylated showing SMPEG in equivalent conditions at the back and b. for variation in PEGylation density by reacting different molar ratios (shown on left) of SMPEG/NH_2 for fixed amine surface desity H.

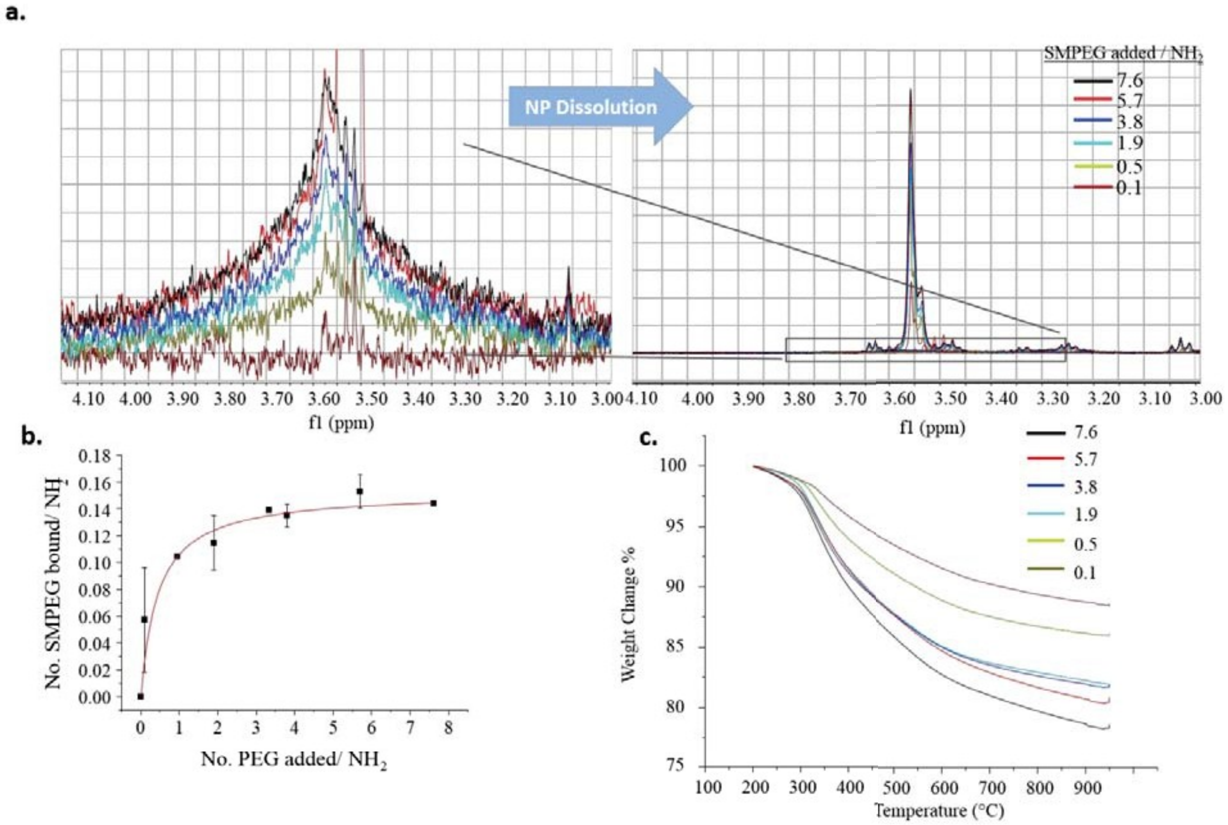

d.

PEG eq. added/NH 2	Nin. Assay	NMR		TGA
	NH_2/nm 2	NH_2 /nm 2	PEG/nm2	PEG/nm2
**5.70**	7.6 ± 1.6	6.7 ± 1	1.16 ± 0.1	1.50
**3.80**	"	"	1.02 ± 0.1	1.36
**1.90**	"	"	0.90 ± 0.2	1.09
**0.95**	"	"	0.79 ± 0.1	0.58
**0.095**		"	0.43 ± 0.3	0.34

Figure s11. ***Varying SMPEG concentration*** **for H type amine density: Representative NP ^{1}H NMR a. pre dissolution and following NP dissolution for NPs corresponding to amine surface density labelled H with varied SMPEG reaction concentrations. b. Combined ^{1}H NMR results for showing variation in SMPEG reaction concentration versus amount bound at the NP surface. c. Representative TGA results for one reaction set. d. Table 2**

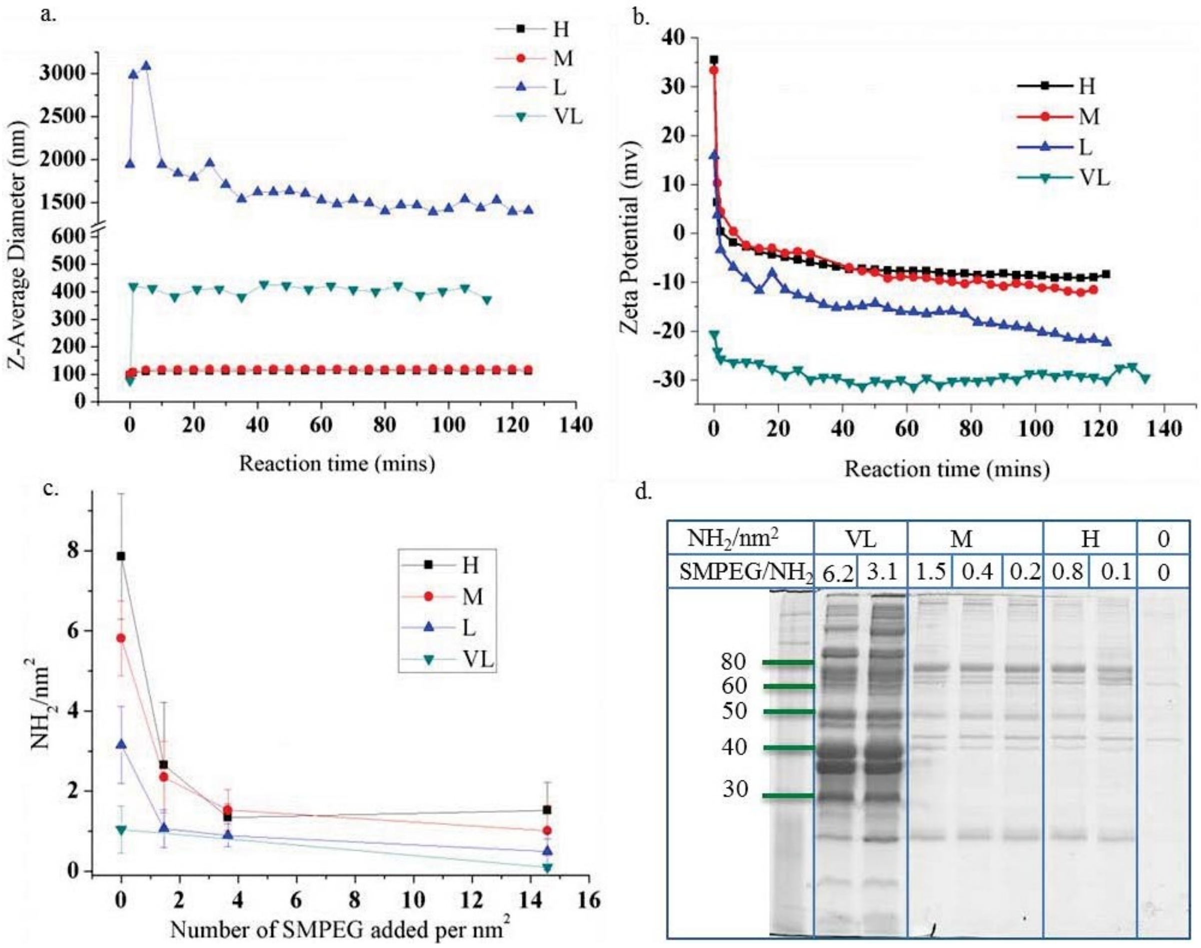

Figure S12. ***Surface Pegylation*** **a. Monitoring the nanoparticle dispersion size by DLS Z-Ave diameter during PEGylation reaction for varied initial surface amine density. b. Corresponding zeta potential measurements during PEGylation reactions. c. Ninhydrin assay results looking at residual amine groups for resulting particles for increasing amounts of PEG linker (per nm^2) added to different amine density NPs H,M,L and VL. d. Protein Corona of PEG particles with varied amine densities and PEG to particle ratios (top) where the 0 sample is the proteins only treated the same way as the particles to obtain a background control.**

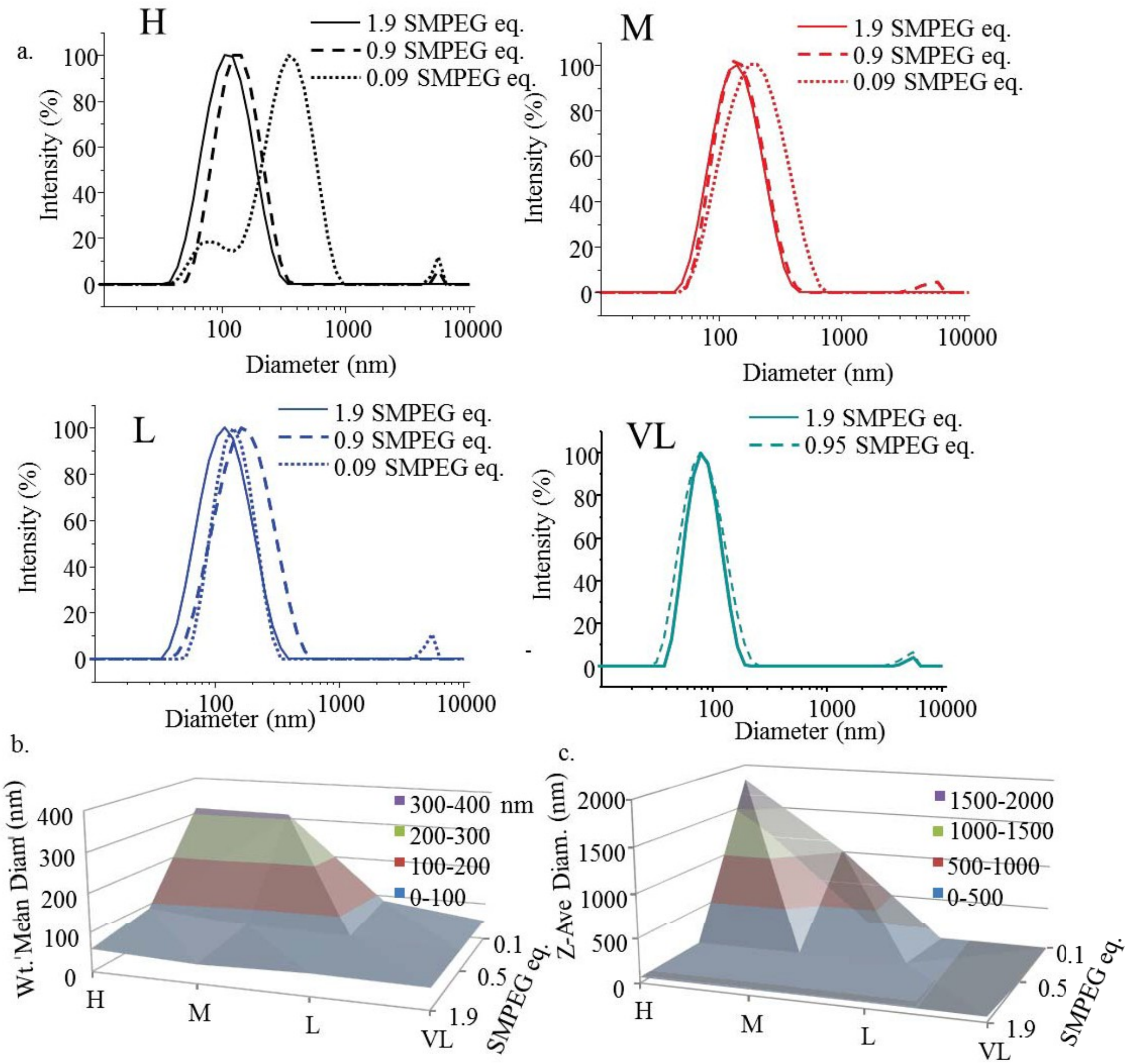

Figure S13. a. Saline PBS Dispersion Studies (DLS and DCS) following PEGylation and centrifugal washing for varied combinations of amine and SMPEG reaction concentrations. Dispersion by intensity obtained by dynamic light scattering of PEGylated particles made with 14.6 (black), 3.6 (red) and 1.5 (blue) PEG molecules per nm^2 added to the reactions for H, M, L, and VL amine particles. b. Composite differential centrifugal sedimentation (DCS) for H, M, L and VL reacted with varied SMPEG amounts and dispersed in PBS c. corresponding dynamic light scattering. All averaged over minimum 3 batches for each point.

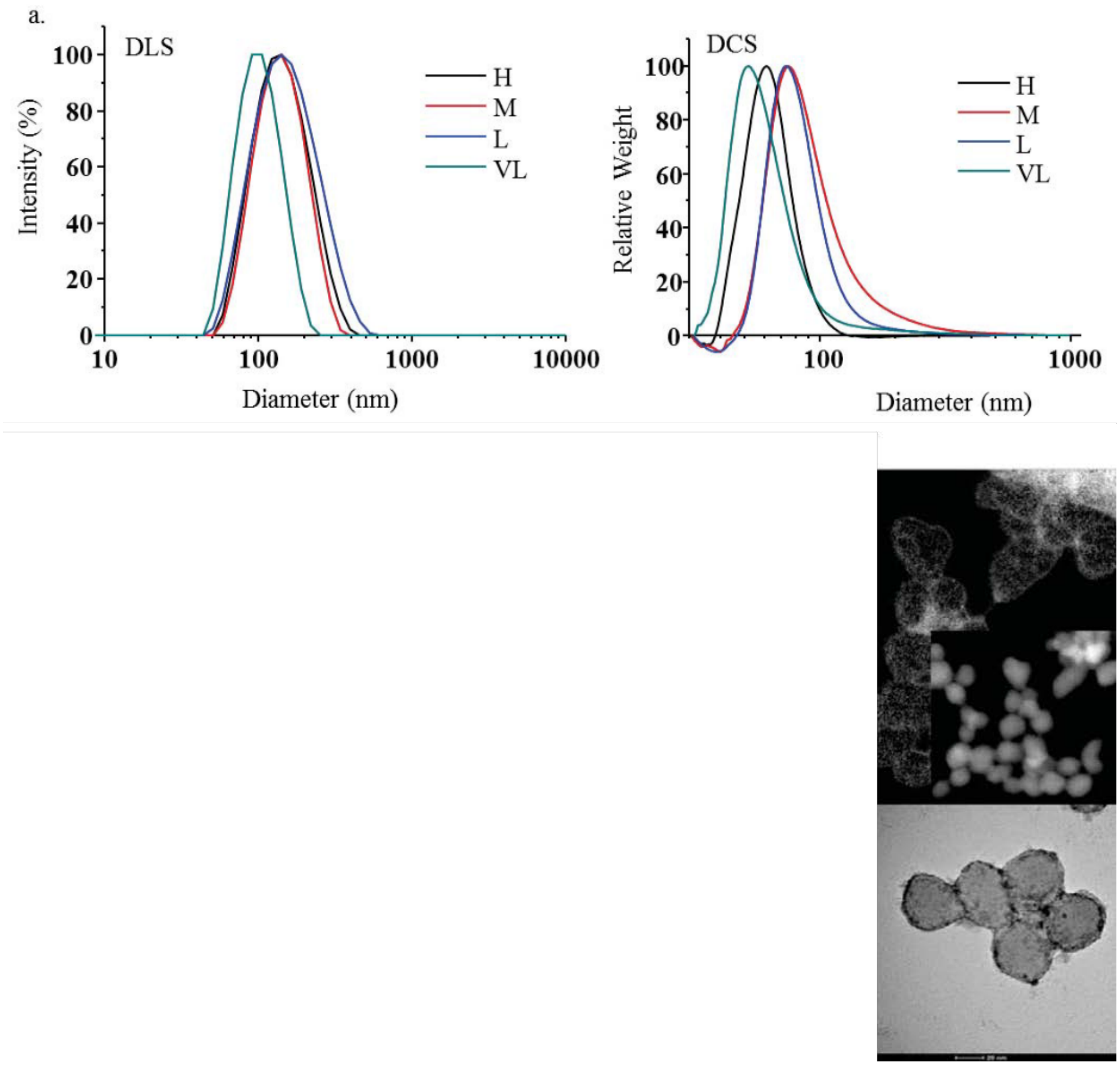

Fig S14. a. Typical dispersion in phosphate buffered saline of H (black), M (red), L (blue) and VL (dark cyan) type PEGylated particles by intensity in DLS and relative weight distribution by DCS. Representative TEM. b. 50 nm FITC- Silica nanoparticles c. E.F.T.E.M. Silicon map of SiO_2-NH_2 (scale bar 20 nm)(inset dark field STEM) d. E.F.T.E.M. carbon map of SiO2-PEG-Mal (inset dark field STEM). e. Uranyl acetate stained T.E.M. of SiO_2-PEG-Tf (H, M, L and VL left to right).

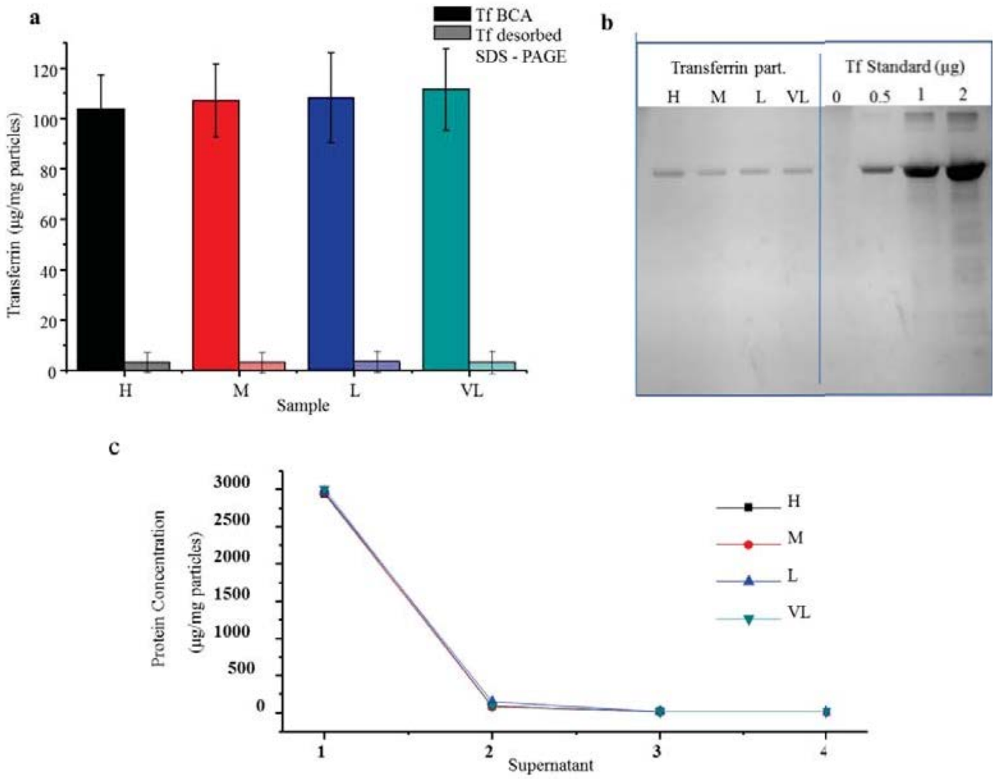

Figure S15. Protein Coupling reaction study a. and b. microBCA and SDS-PAGE study of bound versus unbound protein in reaction mixture. c. Supernatants monitored by BCA for centrigual purification steps following Tf conjugation.

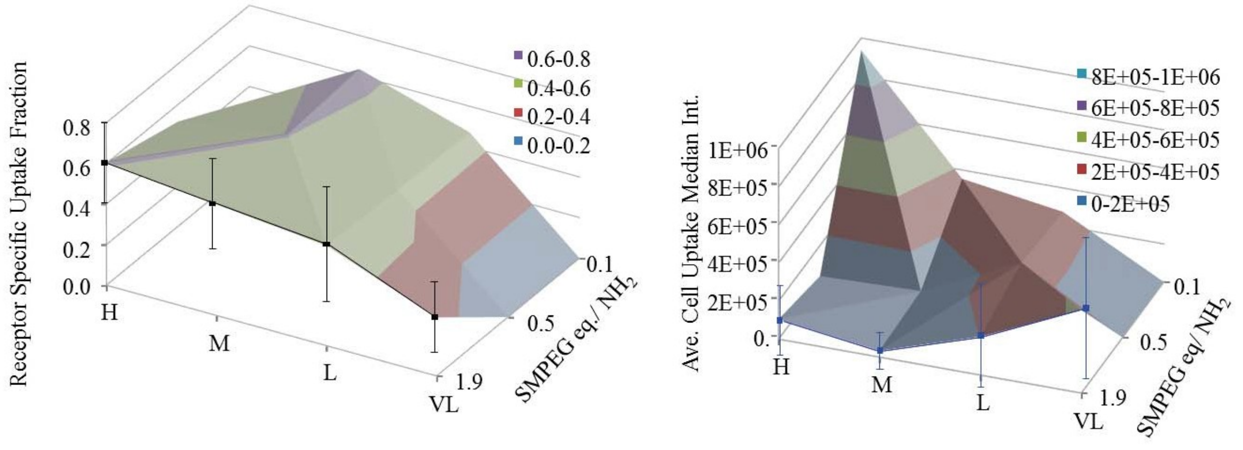

Figure S16. Flow cytometry studies on NP biofunctionality a. Composite (each point minimum three individual batches) of averaged Receptor specific cell uptake fractions (derived from median cell fluorescence intensities) by flow cytometry for synthetic parameter variation and b. corresponding median intensity cell uptake.

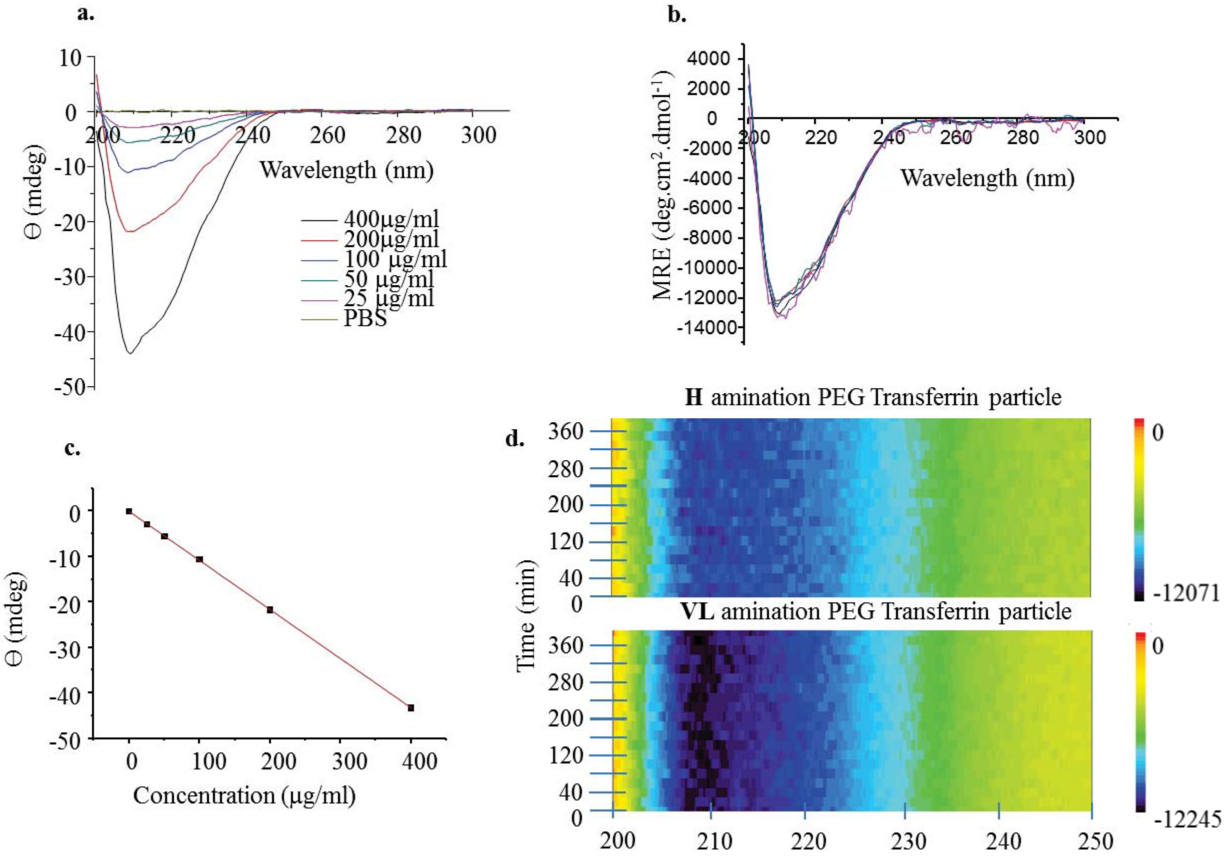

Figure S17. a. Standard curve for Tf concentration determination by CD. b. Mean residue ellipticity normalized calibration curve c. Standard curve d. Time profile CD of H and VL Transferrin grafted silica particles at a concentration of 1 mg/mL, 37°C in PBS for 6 hours.

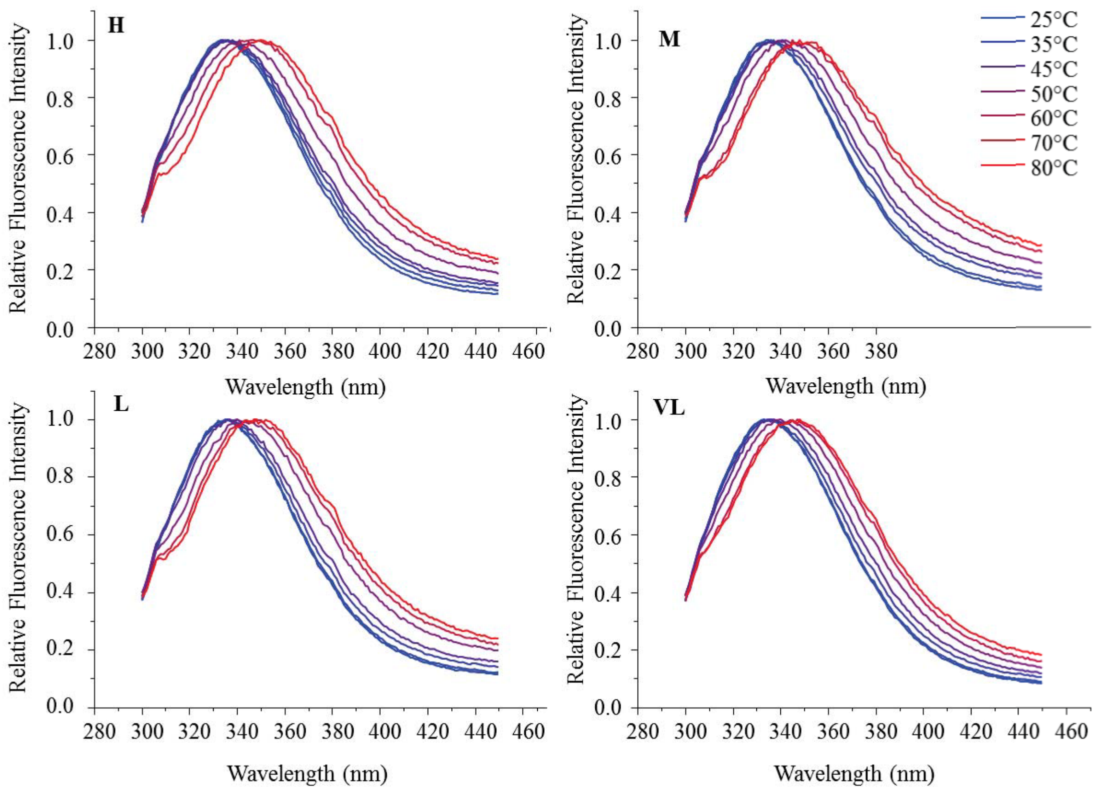

Figure S18. Tryptophan fluorescence temperature - denaturation curves for NPs SiO_2-PEG-Tf H, M, L and VL.

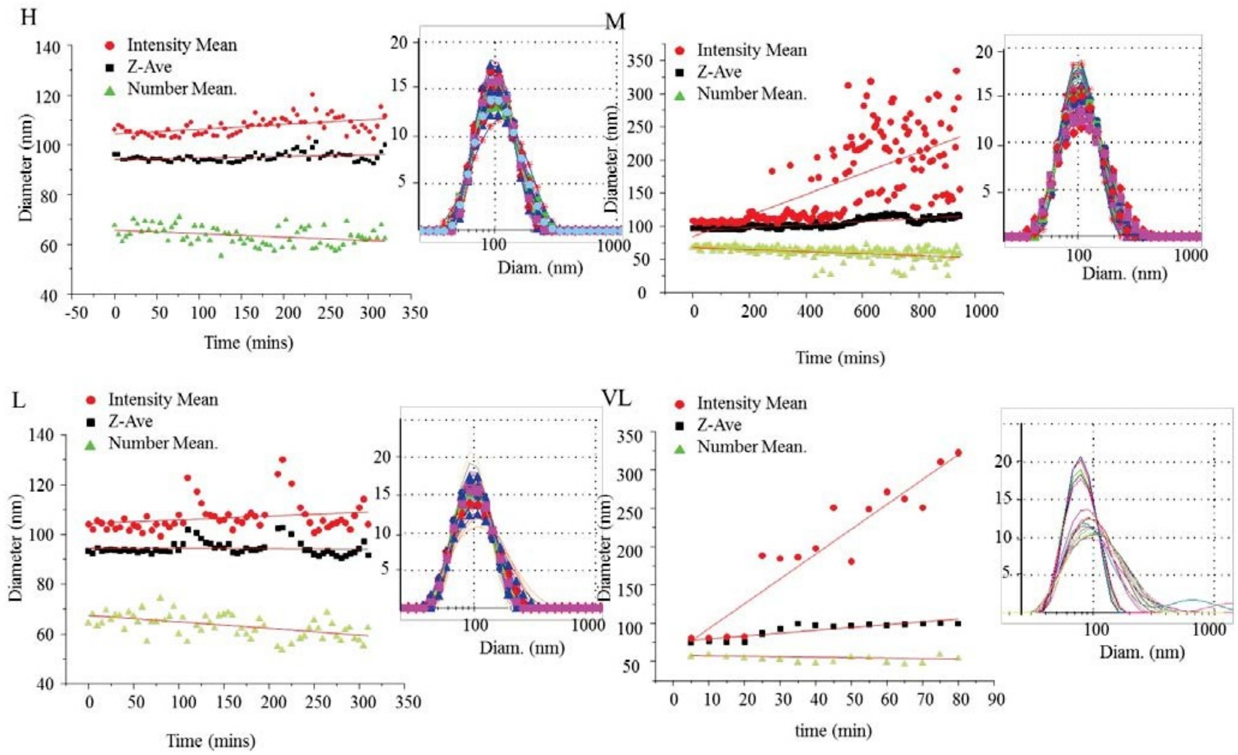

Figure S19. Dispersion Stability in MEM monitored by DLS (Malvern Nano ZS) over course of typical cell uptake experiment time course for varied NP amino function types H, M, L and VL.

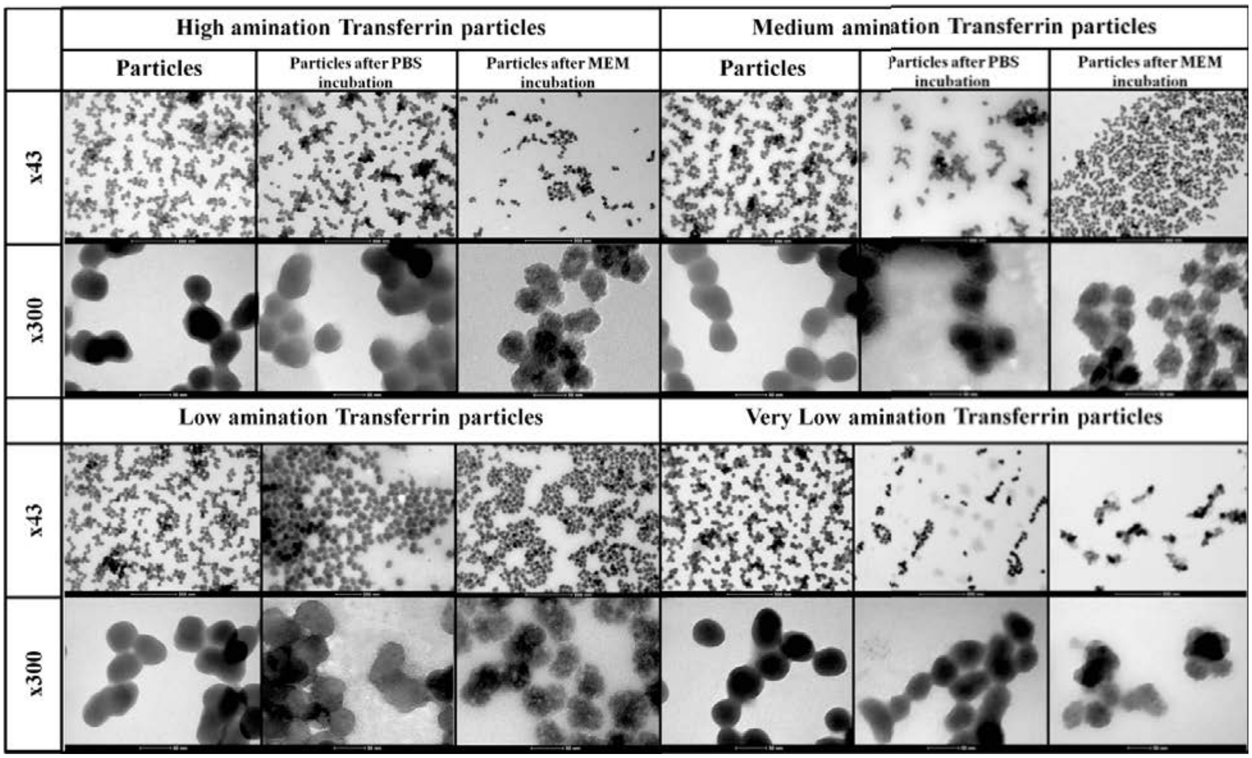

Figure S20. TEM study of structural effects on NPs over course of cell exposure conditions (100 µg/mL 37°C in PBS and serum free MEM) for 6 hours.

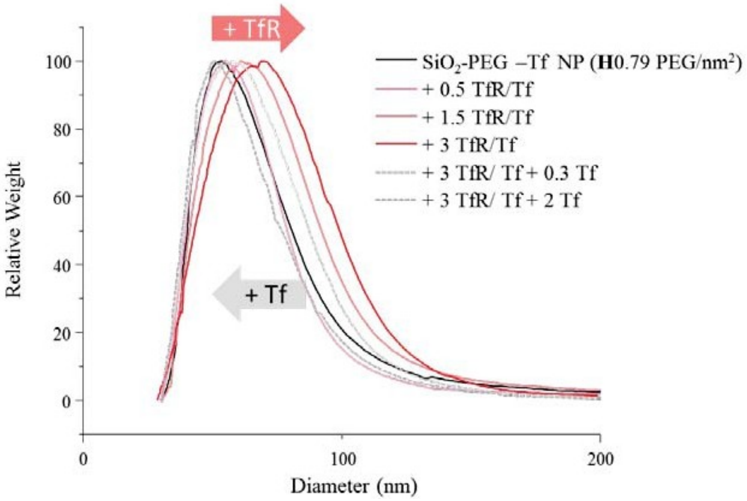

Figure S21. Competitive binding experiment using serum transferrin demonstrating reversibility of receptor sTfR recognition in DCS experiments with maximally functional H0.79 **NPs.**

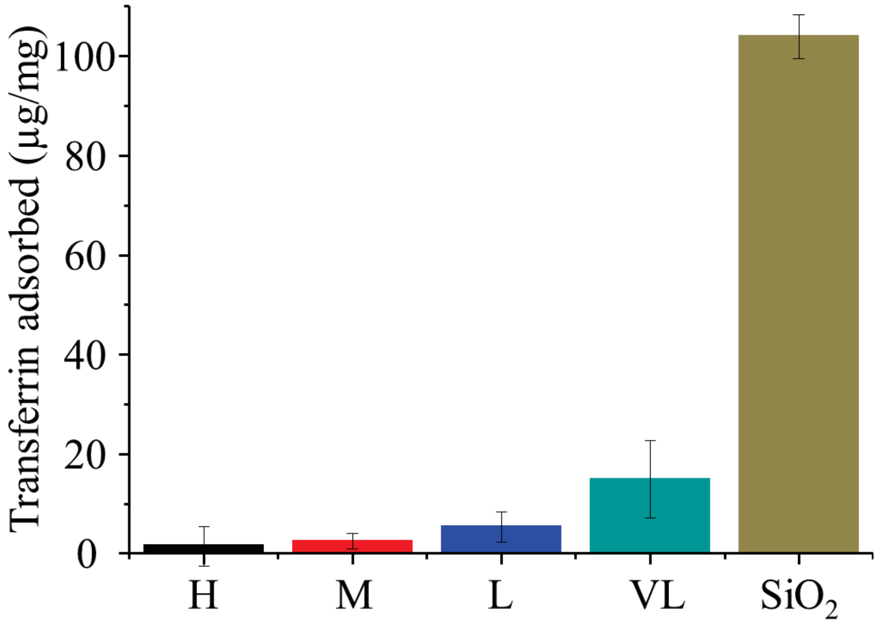

Figure S22. Free Transferrin adsorption on Pegylated particles for H, M, L, VL series measured by BCA (minimum 3 x replicate batches for each type)

Materials and Methods

Materials

All compounds used in the synthesis of the silica core particles were purchased from Sigma – Aldrich. Silica nanoparticles were synthesised as published before[1]. Breifly N-1-(3-trimethoxysilylpropyl)-N'-fluoresceyl thiourea (FITC-APTMS) conjugate solution was prepared by dissolving 4 mg of FITC in 2 ml of anhydrous ethanol. 20 μl of APTMS (11x molar excess) was then added immediately to this solution, with the mixture then shaken at 25°C in darkness for 4 hours. The reaction time course was initially monitored by ^{1}H NMR (CD_3OD).

Silica NP synthesis

All compounds used in the synthesis of the silica core particles were purchased from Sigma – Aldrich. Silica nanoparticles were synthesised as published before[1]. N-1-(3-trimethoxysilylpropyl)-N'-fluoresceyl thiourea (FITC-APTMS) conjugate solution was prepared by dissolving 4 mg of FITC in 2 ml of anhydrous ethanol. 20 μl of APTMS (11x molar excess) was then added immediately to this solution, with the mixture then shaken at room temperature in darkness for 4 hours. The reaction time course was initially monitored by ^{1}H NMR (CD_3OD).

Nanoparticle Preparation

To 25 ml of EtOH (99.9%) was added 0.91 g of NH_4OH (28.0-30.0% NH_3 basis) in a polypropylene container. To this mixture, under rapid stirring, was added 500 µl of the prepared conjugate solution. The reaction was stirred for 15 minutes, upon which TEOS (940 µl) was added. The reaction was then stirred at 600 rpm at 25 °C for further 20 hours in darkness. The resulting nanoparticle suspension was centrifuged down at 14,000 rpm for 20 minutes, with the pellet then resuspended in fresh ethanol aided by bath sonication. This washing procedure was repeated twice more, followed by 3 water washes and a final resuspension in water at a total volume of 12 ml.

Silica Functionalization

Surface Amination

40-45 nm (Weight distribution mean by DCS) particles synthesised and washed were dispersed at a concentration of 10 mg/mL in MilliQ water. Appropriate excess amounts of APTES (e.g. 50 NH_2/nm^2 **H**, 5 NH_2/nm^2 **M**, 1.75 NH_2/nm^2 **L** and 0.4 NH_2/nm^2 **VL**) were added from a 10% v/v ethanolic solution. The calculations were made using the equation in the following section. The final solution pH was measured at 10 and the particles are shaken at 800 rpm and 25°C for one hour. The temperature was then increased to 90°C and the suspension was shaken for one hour more. The resulting particle dispersion were washed by centrifugation and resuspension with water (x4) and finally re-dispersed in water at a concentration of 10 mg/mL. Surface amine density was measured by Ninhydrin assay and dissolution ^{1}H NMR.

Protein grafting onto nanoparticles

All compounds used were purchased from Sigma – Aldrich unless stated otherwise. All buffers used were prepared just prior to use, degassed by bubbling with nitrogen for 20 min at a moderate rate and filtered through a syringe filter (pore size 0.2 µm) before use.

Step 1: PEGylation of NH_2 functionalized silica nanoparticles

HEPES buffer(20mM pH7.4) was used for the PEGylation reaction prepared by dissolving 1.19 g of HEPES (4-(2-hydroxyethyl)-1-piperazineethanesulfonic acid) in 250 mL of MiliQ water yielding a final and then adjusting to pH 7.4 ± 0.05 using 5M NaOH. The buffer was deoxygenated by bubbling with nitrogen. Amino functionalized NPs were washed twice with Hepes buffer (20 mM pH7.4) with redispersion in the same buffer at a final concentration was 10 mg/mL. Succinimidyl-([*N* maleimidoproprionamido]-octylethyleneglycol)ester (SM(PEG)8) (Thermo Scientific) was diluted in HEPES immediately prior to mixing with NP dispersion at a 1 to 1 volumetric ratio giving a final NP reaction concentration of 5 mg/ mL. The mixture was left shaking at 700 rpm at 25°C for 2 hours. Particles were washed twice with the HEPES buffer and then re-dispersed in the same at a concentration 10 mg/mL.

Step 2: Modification of the protein (Transferrin) with $SATPEG_4$

Holo – Transferrin was dispersed in PBS pH 7.4 (5 mg/ mL, 62.5 PM) and to it N-Succinimidyl S-acetyl(

thiotetraethylene glycol) SAT(PEG)$_4$ (Thermo Scientific) was added in a 1:1 molar ratio with Transferrin, the solution was left slowly shaking at 400 rpm, at RT for 30 min after which time a deacetylation solution was added (0.1 mL/mL reaction) and was left to react for a further 2 hours.

The deacetylation solution was made as 348 mg Hydroxylamine (0.5 M) and 73mg of disodium salt EDTA (25 mM) dissolved in 9 mL PBS with the pH then adjusted with 5M NaOH to 7.4.

Following this modification purification was performed using pre-packed NAP 25 sephadex (GE Healthcare) columns which were pre equilibrated with Hepes 7.4 for three column volumes. 1 mL of the modified protein solution was passed through. The protein was collected (if multiple columns were used all of it was pooled together) and tris(2-carboxyethyl)phosphine (TCEP) (1:1 TCEP to Transferrin molar ratio) was added. Modified protein was combined with the PEG-Maleimide in less than 15 minutes following purification in all cases.

Step 3: NP-Protein conjugation

The protein was put in a polypropylene tube (Eppendorf LoBind®) and was combined with NP dispersion to give a final mass ratio of 1:1. The dispersion mixture was then left for 2 hours at 400 rpm shaking at room temperature. 2 – mercaptoethanol was added to the dispersion (1 mM final concentration) which was then left for 5 minutes after which particles were washed (12,000 rpm for 15 minutes, redispersion by inversion followed by 5 seconds bath sonication) four times with PBS pH7.4 and finally re-dispersed in PBS pH7.4 at a concentration of 10 mg/mL. For final redispersion pipetting is simultaneously applied with bath sonication.

Optimisation of reaction conditions Particles were synthesised as above however several variations were introduced. The purified protein (after step 3) was separated in two fractions, TCEP (1:1 molar ratio to Tf) was added to one but not the other. After the end of the protein conjugation (beginning of step 5) the particles were split into two, 2 – mercaptoethanol (1 mM) was added to one but not the other. This yielded four protein batches.

Nanoparticle Characterization

Dynamic light scattering and zeta potential

A Malvern Zetasizer ZS series with an autotitrator accessory was used in all measurements. 10 µL nanoparticle dispersion samples, at a concentration of 10 mg/mL, were taken and dispersed in 990 µL 1mM NaCl or PBS in a plastic low volume cuvette (PLASTIBRAND, semi-micro, PMMA, l = 1 cm) giving a final concentration was 0.1 mg/mL. Particles were measured twice, both measurements consisted of two manual measurements each eleven runs for a total of forty four accumulations. The number presented is an average of those measurements. After size measurement particles were transferred to a zeta potential cuvette (disposable capillary zeta cell) and measured twice using two manual measurements with eleven runs each for a total of forty four accumulations. All measurements used the Smoluchowski model. The zeta potential presented is an average of those measurements. For Zeta potential measurements the

conductivity was always above 0.1 mS/cm for 1mM NaCl and > 14 mS/cm for PBS measurements. pH of all dispersant solutions was 7 unless specified otherwise.

Monitoring of SMPEG –NP Reaction using dynamic light scattering

Size

0.5 mL of amine functionalized particles at a concentration of 10 mg/mL in HEPES 7.4 were taken and put in a low volume (PLASTIBRAND, semi-micro, PMMA, l = 1 cm) plastic cuvette and their size was measured, this was used as the 0 hour time point. The particles were taken out of the cuvette and 0.5 mL of PEG solution in HEPES 7.4 at the appropriate concentration was added. Dispersion was placed in a plastic cuvette (l = 1 cm) and a continuous measurement was started. Set up was such that the instrument makes a measurement every five minutes for two hours, every measurement consisted of eleven runs. The attenuator was fixed to 5 and the count rate was monitored, it didn't alter significantly across the course of the measurements. Particles were homogenized once every half an hour using pipetting. For the size kinetics of particles with Low amine density (~1 NH2/nm2) homogenization by pipetting was required before each measurement as they tended to aggregate with some settling.

Zeta potential

0.7 mL of amine functionalized particles at a concentration of 10 mg/mL in HEPES 7.4 were taken and put in a disposable capillary zeta cell and their zeta potential was measured, this measurement was used as the 0 hour. The particles were then take out of the cuvette and 0.3 mL of PEG solution in HEPES 7.4 at the appropriate concentration was added, the dispersion was homogenized and put back in the same cuvette. A continuous measurement was set up as follows: a measurement was made every five minutes, each measurement consisted of eleven runs. The total time of the study was two hours.

Autotitration in the DLS

The pH meter on the Malvern autotitratior was calibrated at pH 4 and 7 using pH standards. Milli-Q water was then flushed through the sample loading tubes of the instrument for 10 cycles. The tubes of the titrator were primed with 0.1M NaOH for five cycles. 100 µL of particles (10 mg/mL) were taken, put in an 2 mL polypropylene tube, and to them, 10 µL of 1M HCl was added, this dispersion was bath sonicated for 10 minutes. It was diluted in 9.9 mL Milli-Q water for a final volume of 10 mL. A standard autotitration container (25 mL polypropylene tube) was used in the setup with a magnetic stirrer. Sample dispersion was then flushed through the sample loading tubes for five cycles. Final pH of the system was typically 2.8 ± 0.2 and initial conductivity (measured by DLS) was 0.5 (mS/cm).

A zeta potential and size measurement were taken every 0.2 ± 0.1 pH units, both types of measurement were set up as automatic. Continuous stirring was applied to ensure a homogeneous dispersion.

Following the experiment, water was flushed through all the tubes, both the sample loading and the titrator, for thirty cycles changing the direction every ten cycles.

Timecourse monitoring of Tf grafted particles using DLS

Particles were dispersed at a concentration of 0.1 mg/mL as 10 µL of particles (10 mg/mL) were diluted with 990 µL of serum free cell medium (SF) I = 0.15M. Samples were placed in a large volume quartz QS cuvette (l = 1 cm) which was sealed with a plastic stopper, the cuvette was placed in the instrument and the temperature of the sample holder was adjusted to 37°C.

A size measurement was made once every five minutes, each measurement consisted of eleven runs.

Differential centrifugal sedimentation (DCS)

DCS was performed using a CPS Disk Centrifuge DC 24 000. 30 µL of clean particles at a concentration of 10 mg/mL were taken and dispersed in 70 µL of PBS for a final concentration of 2 mg/mL. The disc speed of 18 500 rpm was used and an 8% - 24% PBS based sucrose gradient was injected (settings optimized for size range analysis 0.03 – 1 µm). A 476 nm PVC (Analytik UK) commercial standard was used to calibrate the instrument before each measurement. Each gradient was checked by running the PVC standard as a sample and comparing to a database control. 100 µL of standard was injected before each measurement to calibrate the instrument.

Transmission electron microscopy (TEM)

TEM of SiO_2 particles

Silica particles were diluted to a final concentration of 0.1 mg/mL with water and 10 µL were transferred on a Formvar carbon 400 mesh copper TEM grid (Agar Scientific) and left to dry in air overnight. The grid was imaged using FEI Tecnai 120 instrument using 120keV.

Scanning Transmission Electron Microscopy (STEM), elemental maps of silica and silica PEG particles using Energy Filtered Transmission Electron Microscopy (EFTEM)

SiO_2 and SiO_2 – PEG particles were diluted to a final concentration of 0.1 mg/mL with water and 10 µL were transferred to a lacey carbon grid and left to dry in a desiccator for 1 hour. Bright and dark field STEM and EFTEM were done using a FEI Titan 80–300 (aberration corrected for TEM imaging) instrument without any staining using 300keV.

Staining procedure and TEM of SiO_2 – PEG particles

Particles were diluted to a concentration of 0.1 mg/mL with water and 10 µL were transferred to a 400 mesh copper TEM grid. The drop was left for 10 minutes after which the grid was washed 3x with drops of H_2O and left to fix with 2.5% glutaraldehyde for 10 minutes. The grids were then washed 5x with drops of H_2O and put on a contrast solution of 2% urenyl acetate for 15 minutes. Grids were rinsed quickly on another two drops of H_2O and put in a solution of 0.13% methyl cellulose 0.4% uranyl acetate for about 5 minutes to create a thin polymer layer over the particles. Grids were then taken out of the solution using a wire loop and the excess methyl cellulose was removed carefully with clean filter paper. Finally the grids were left to air dry and imaged on a FEI Tecnai G2 20 Twin instrument using 200keV.

Ninhydrin assay

Establishing a calibration protocol

To determine the suitability of APTES as a calibration compound it was compared to other standard amines, octylamine and propylamine. Solutions of concentration 5 mM were prepared in absolute ethanol and serially diluted to generate calibration curves. The highest and lowest concentrations were 5 mM and 0.325 mM respectively.

Analysis of amine functional groups on nanoparticle surface

0.1 ml of washed amine particles and a bare silica control were taken and put in a Safe Lock® eppendorf tube to prevent evaporation at high temperatures. Particles were spun at 14 000 rpm for 10 minutes, the water was removed carefully using a pipette and 1 mL of dry ethanol was added, particles were dispersed using sonication, and the process was repeated twice more using 1 mL of dry ethanol before being finally redispersed in 0.5 mL of dry ethanol. 5 mM solution APTES was made and diluted five times by a factor of two to produce a calibration, dry ethanol was used as a blank. A fresh ninhydrin solution of concentration 3.5 mg/mL (20 mM) in dry ethanol was prepared before each experiment. 0.125 mL of the solution was added to each of the analysed samples (0.5 mL each), the Safe Lock® Eppendorf tubes were closed and put at 60ºC for 40 – 45 minutes. After that time the dispersion had a blue/purple colour. The particles were spun out of it and 0.2 mL of the supernatant was taken with a pipette and placed on a multiwell plate. Their absorption was analysed using a plate reader at 566 nm.

Fluorescence measurements

5 µL of clean particles at a concentration of 10 mg/mL were taken and dispersed in 495 µL of PBS, of that 100 µL was taken and put in a low volume quartz cuvette (Hellma® fluorescence cuvettes, ultra Micro) and the emission spectra (from 500 – 600 nm) measured in a Fluorolog 3 at an excitation of 480 nm using 2 nm slits widths.

Determining the concentration by ultra – balance weighing

A Sartorius Ultramicro balance was calibrated internally before use, standard weights (1 mg, 200 mg and 2000 mg (Sartorius YCW0121-00)) were used to confirm calibration and to establish accuracy of balance. 500 µL LoBind Eppendorf polypropylene tubes were pre-weighed before adding 50 µL of nanoparticle dispersion. The liquid was removed using a rotor evaporator (Eppendorf Concentrator Plus) for over 7 hours. The tubes containing with the dry powder were taken out of the rotor evaporator and left to equilibrate with room temperature for a minimum of four hours. Control weights for solution containing buffers and salts etc. were obtained by drying equal volumes of such solutions. Weights presented are an average of three measurements.

Characterization of grafted Transferrin

Micro BCA assay

Commercially available Micro BCA assay kit (Thermo Scientific) was used for determining the protein concentration determined against a provided Bovine serum albumin (BSA) standard curve.

Circular Dichroism

A JASCO J.810 was used for all circular dichroism (CD) measurements. A 1 mm quartz QS cuvette was used for all CD studies in conjunction with a spacer (total width was 1 cm). Before each use the CD

cuvette was rinsed using PBS, dried and 200 µL of PBS 7.4 was put in and used to background the instrument. PBS was run as a sample to confirm the background was correct. CD was set up so that all graphs are taken as the average of eight runs at a rate of 50 nm/min and each presented CD spectra is the average of a minimum of two such experiments. The cuvette was washed with PBS between measurements. Particles were taken out of storage, homogenized using pipetting and their concentration was adjusted to 1 mg/mL, 150 µL of dispersion was pipetted into the cuvette. The CD measurement was then conducted at the same conditions as the background. For mean residue ellipticity (MRE) normalisation of spectra protein concentration was calculated from CD standard curves.

Time-Stability study using circular dichroism

The sample preparation was the same as used for a single CD measurement. A Peltek unit was set to 37эC and the system was left to equilibrate for a few minutes. A single PBS measurement (8 scans 50 nm/min) was conducted to background the instrument.

The particles were then placed in the cuvette, it was sealed and placed in the CD. The time lapsed measurement consisted of a set of twenty single measurements, as were described above, with no waiting time in-between. A 2D spectra was then obtained from the instrument software.

Protein concentration determination by CD

All samples were measured as described above. A standard curve was prepared using Transferrin of concentration 35 µM determined by UV-Vis (extinction coeff. 114 000 $M^{-1}cm^{-1}$ at 280 nm) which was serially diluted to yield 17 µM, 8.65 µM, 4.3 µM and 2 µM. Protein solutions were then measured as described above. The signal max at 210 nm was taken and a calibration curve was plotted using ellipticity (mdeg). To verify the curve the spectra were then plotted in MRE and an overlap was observed.

SDS-Page for determination of adsorbed protein on the surface of Tf conjugated nanoparticles

For each NP sample, the total amount of 0.1 mg of Tf grafted silica nanoparticles was resuspended in 10 ȝO RI 3%6. Similarly, the Tf samples (for calibration curve) were prepared by resuspending varying amount of Tf (from 0 to 2 ȝJ) LQ 10 ȝ/ RI 3%6. $OO WKH VDPSOHV ZHUH ERLOHG IRU 5 PLQXWHV LQ loading buffer (62.5 mM Tris-HCl (pH 6.8 at 25 °C), 2 % (w/v) sodium dodecyl sulfate (SDS), 10 % glycerol, 0.01 % (w/v) bromophenol blue, 40 mM dithiothreitol (DTT)) (New England BioLabs) and separated by size of the moiety of porous 10% polyacrylamide gel in an electric field, as described below. The gels were stained using 2D Silver Stain Kit II (Cosmo Bio Co., Ltd) and scanned using Bio-Rad GS-800 Calibrated Densitometer. Subsequently, TotalLab Quant software has been used to perform densitometry on the bands and calculate approximate amounts of free Tf per mg of nanoparticles.

SDS – PAGE

Two glasses, one to hold the gel and one to cover it with a 1 mm distance between them are used to make the gel frame. They're cleaned by a surfactant wash, an ethanol wash and finally rinsed with deionised water and dried well with a paper towel so that no lint or water is left on the glass.

For two gels: 10% SDS – PAGE gel was precast fresh before each experiment as 3 mL of Milli-Q water, 3.75 mL of 1.5 M Tris – HCl buffer pH 8.8 ± 0.1 and 0.1 mL of 10% in water sodium dodecyl sulphate were

mixed well and to them 2.5 mL of 30% in water acrylamide. 5 µL of Tetramethylethylenediamine (TEMED) were added and the solution was mixed again, 50 µL of ammonium persulfate 10% by weight in water was added last. Gel solution was poured in the frame, 0.5 mL of 2 – Propanol was pipetted on top of it to prevent evaporation and the gel was allowed to set for about 20 minutes.

While the gel was setting a second, 4% stacking gel was made: 2.82 mL of Milli-Q water, 1.575 mL of 0.4 M Tris – HCl buffer pH 6.8 ± 0.1 and 50 µL of 10% in water sodium dodecyl sulphate were mixed well and to them 0.5 mL of 30% in water acrilamide and 5 µL of Tetramethylethylenediamine (TEMED) were added. The solution was mixed again. Before the 50 µL of ammonium persulfate 10% by weight in water was added. The 2 – Propanol was removed from the top of the 10% gels. The full stacking gel solution was poured on top of the 10% gel and a 10 well 1 mm come was put in it to make the wells. The gel was left to set for another 15 minutes.

10 µL of clean particles at a concentration of 10 mg/mL were taken and to them a commercially available loading buffer (as above) and DTT solution were added. Particles were boiled at 100°C for 5 min and 20 µL/well were added and gel was run at 130 V for about an hour.

Silver staining of Gels

Gels were taken out of the frame carefully and put in a fixing solution (Fix 1) containing 50% Milli-Q water, 40% Methanol and 10% Acetic acid for at least one hour.

Silver staining was done using a commercially available kit (InsightBio) and consists of six solutions (1 – 6) which are used in succession with a series of washing steps. The following fixes are used:

Fix 2: 55% Milli-Q water, 30% methanol, 10% acetic acid and 5% solution 1

Fix 3: 45% Milli-Q water, 50% methanol and 5% solution 2

Fix 4: 90% Milli-Q water, 5% solution 3 and 5% solution 4

Fix 5: 95% Milli-Q water and 5% solution 5

Fix 1 was carefully decanted off and fix 2 was poured with the gel, it was left for 15 minutes, fix 2 was then decanted and fix 3 added, gel was left for 10 minutes. Fix 3 was decanted off and the gel was washed with Milli-Q water for 5 minutes after which fix 4 was added and system was left an additional 15 minutes. Fix 4 was removed and at which point the gel was washed with Milli-Q water three times for 2 minutes each. The development solution, fix 5 was added and the gel was stirred by hand until bands were observed. When bands were significantly developed 2.5 mL of solution 6 was added. Gel was washed with Milli-Q water twice. Unless specified otherwise all fixes were added as 50 mL/gel and while waiting were stirred at 40 rpm on a gel shaker.

Thermogravimetric Analysis

All thermogravimetric analysis were done using a Q500 from TA instruments. 1 mL of PEG functionalized particles (approx. 10 mg/ mL) were washed twice with Milli-Q water, suspended in Milli-Q water, put in an Eppendorf polypropylene tube and dried using a rotating evaporator (spinning at 1400 rpm with a temperature of 60эC and under vacuum) overnight. Dry pellet was obtained. Air and nitrogen were used as the blowthrough gas (90 mL/min) and balance gas (10 mL/min), respectively. A platinum sample holder was rinsed with water, acetone and dried. The gasses were turned on and the instrument tared. Sample was then put on the sample holder and loaded into the instrument prior to starting a measurement. The

sample was heated up at a rate of 20эC/min until the temperature reached 150эC and left for 45 minutes to remove any leftover moisture. Temperature was then further increased to 950эC at a rate of 25эC/min. Evolved gasses were put through a mass spec and the compounds with a molar mass of 18 Da for water and 44 Da for carbon dioxide were monitored.

Particle dissolution studies in DLS

Core silica particles were placed in a plastic cuvette (l = 1 cm) at a concentration of 10 mg/mL and NaOH was added so that the final concentration was 50 mM, 100 mM and 200 mM. The cuvette was sealed well with parafilm and placed in the instrument where a size measurement was taken every 5 minutes for twelve hours (temperature was set to 37эC) with a fixed attenuator of 11. The drop in count rate over time was observed. In this way the standard particle dissolution procedure was established. Particles were dispersed in 200 mM NaOH in a Safe Lock® Eppendorf and left 16 hrs at 37эC. Particle dissolution is then confirmed using a standard DLS measurement (section 3.1.2).

Particle dissolution studies using SDS – PAGE

Silica amine functionalized particles were dispersed at a concentration of 10 mg/mL in 200 mM NaOH in a Safe Lock® Eppendorf and left to incubate for several time points: 3, 6 and 24 hours at 37эC.
15 µL of the particle dispersion were taken and loaded on a SDS – PAGE (made as described below) and gel was run at 130 V for 30 minutes. Plates were taken off the rack and scanned fluorescently. Experiment was repeated three times.

Gel Fluorescence scanning

The in-frame gel was taken and scanned for FITC using the automated settings of the Syngene G:Box fluorescent gel scanner: blue LED and a 525 nm filter.

Nuclear Magnetic Resonance

In all cases a 5 mm thin wall, 8 inch, 500 MHz NMR tube was used (Wilmad Lab Glass). Oxford instruments 400MHz and Varian 600MHz instruments were used, measurements were conducted at 25°C with no equilibration time and 45° detection angle.

Determining the T1 using ^1H NMR

All T1 relaxation times were determined using a standard Inversion-recovery experiment run on a Varian 600 MHz NMR.

APTES and SMPEG8 calibration curves

APTES at a concentration of 11.7 mg/mL (50 mM), 5.53 mg/mL (25 mM), 2.21 mg/mL (10 mM), 1.11 mg/mL (5 mM), 0.5 mg/mL (2.25 mM) and 0.1 mg/mL (0.45 mM) was dissolved a 1 mL solution of 200 mM NaOD with 1mM DMF as an internal standard. The solution was left overnight at 37°C to match dissolution conditions and then measured. Accumulation time in the measurement was 10 seconds and each measurement consisted of 32 scans. Six calibration curves were measured in a 400 MHz NMR and one in

a 600 MHz instrument. Averaged, the three peaks corresponding to the (C-H) on the propyl chain were used to determine concentrations for the calibration curve.

SM(PEG)$_8$ at a concentration of 11.6 mM, 10 mM, 8 mM, 5.8 mM, 5 mM, 4 mM, 2.9 mM, 2.5 mM, 2 mM, 1.5 mM, 1.25 mM, 1 mM, 0.725 mM, 0.625 mM, 0.5 mM, 0.25 mM and 0.15 mM was dispersed in a 200 mM NaOH solution with 1 mM DMF as an internal standard. The solutions were left overnight at 37°C and measured in a 400 MHz and 600 MHz NMR for 32 scans with an accumulation time of 10 seconds. The composite peak from the ethylene oxide backbone (at 3.6 ppm) was used for the calibration.

^{1}H NMR measurements of the PEG on intact particles

1 ml of particles of known concentration were washed twice with D_2O and finally re-dispersed in 1 mL, 1mM solution of DMF in D_2O. Two measurements were made: first a short measurement (8scans 10 seconds accumulation time) to check that impurities, such as ethanol or acetone left over from washing the NMR tube, were not present. If so the samples was taken out, washed twice more and re-measured using a 400 MHz NMR (128 scans and 10 seconds accumulation time).

Dissolution ^{1}H NMR

Following the above described measurements on intact nanoparticles, an aliquot of NaOD (5M in D_2O) was then added so that the final concentration is 0.2 M. The dissolution procedure was as described above i.e. incubation for 16 hrs at 37϶C

The dissolved particles were put in a clean NMR tube and measured in a 400 MHz NMR (16 scans 10 seconds accumulation time).

Analysis of APTES-silica NP reaction and purification by ^{1}H NMR

The appropriate amount of Silica core nanoparticles was taken and washed into D_2O three times to completely remove the water. The reaction was then carried out as described above.

After the reaction, 700 µl was kept aside for characterisation using NMR. The rest of the particles (**H**, **M**, **L** and **VL**) were moved to eppendorf tubes and spun down at 14 000 rpm for 20 minutes. The supernatant was removed using a plastic pipette and kept aside while the particles pellet was re-dispersed using D_2O. The particles were pooled together into a 15 ml falcon tube and further 700µl was kept aside. The rest of the particles (**H**, **M**, **L** and **VL**) were moved into eppendorf tubes and spun down and the process was repeated again for further 3 more washes.

The solutions are loaded in NMR tubes and scanned in a 400 MHz instrument (8 scans, 10 second accumulation time). Supernatant obtained after each wash step were also measured using the same procedure.

NMR spectra Processsing

MestReNova 8.0 software was used for chemical shift predictions, Lorentz fitting of peaks for FWHM determination and integration of peak signal. All NMR spectra were processed in the following fashon: the obtained spectra was chemical shift referenced using the internal standard (DMF) and standard solvent peak, an exponential apodization of 0.3 Hz was then applied, the phase of the spectra was corrected manually (if required). Spectra were then baselined using Polynomial Fit in all cases. Peak picking,

integration and peak fitting were done in manual mode using MNova software. Peak fits were compared between MNova and exported into Origin8 using a Lorenz fitting protocol.

Particle stability in biological media

Particles were dispersed in PBS or MEM so that the final concentration was 0.1 mg/mL and left stationary at 37°C for 6 hours. The particle dispersion was characterized using TEM.
10 µL of the as obtained dispersions was pipetted on a Formvar carbon 400 mesh copper TEM grid (Agar Scientific) and left to dry overnight. TEM (Tecnai 12 at 120 keV) images was taken at several magnifications.

Characterization of interaction with proteins

Protein corona

Hard protein coronas on NPs were prepared by incubating the nanoparticles at a concentration of 0.5 mg/ml in ~100% human plasma (total sample volume of 0.5 ml), for 1 hour at 25°C. After the incubation, samples were spun down for 30 min at 18 000 rcf and washed with 0.5 ml PBS using pipetting. The three cycles of washing steps allow for removal of proteins loosely bound to NPs surface (so-called soft corona) with recovering of the so-called hard corona composed of the most affine proteins. The final pellet was resXVSHQGHG LQ 20 ȝO RI 3%6. The nanoparticle – protein complexes were separated and denatured by boiling for 5 minutes in loading buffer (62.5 mM Tris-HCl (pH 6.8 at 25 °C), 2 % (w/v) sodium dodecyl sulfate (SDS), 10 % glycerol, 0.01 % (w/v) bromophenol blue, 40 mM dithiothreitol (DTT)). Samples were then separated by size in the moiety of porous 10% polyacrylamide gel as described above.

Protein adsorption study

1 mg of particles were washed into PBS 7.4 twice and redispersed at a concentration of 2 mg/mL in 0.5 mL of PBS pH 7.4. To these 0.5 mL of 5 mg/mL Bovine Serum Albumin was added and the dispersion was left to equilibrate for an hour at 37ºC. The as made dispersions were spun at 13 000 rpm for 15 minutes and resuspended in pure PBS 7.4 using pipetting. Three more washes followed. Finally the particles were dispersed in PBS 7.4 and the protein content on the surface was measured by microBCA assay.

Biological Interaction characterization

Receptor binding studies

In order to titrate the silica nanoparticles with Transferrin receptor (TfR) (R&D Systems) it was vital to ensure that the colloidal stability of the particles did not vary from batch to batch, whilst this is true for most cases, in some low degrees of agglomeration made meaningful titrations difficult to obtain. To overcome this, 0.1% (wt/wt) Bovine Serum Albumin (BSA) was introduced across all samples as a means of increasing the consistency in colloidal stability of the system across batches.
Lyophilised transferrin receptor aliquots were reconstituted by gently warming the vial to room temperature and adding 250µL in PBS 7.4 which had been filtered through a 0.22µm filter. In a typical titration, stock nanoparticles of Transferrin grafted silica (10mg/mL) were briefly sonicated for ca 5s. 20µL of stock was

removed and placed in a separate Eppendorf, to this 10μL of 1%(wt/wt) BSA was added. The particles and BSA were incubated for 5 mins at room temperature. After this incubation the particles were diluted to a final volume of 100 μL with either PBS or a PBS TfR mixture. The final concentration of particles, BSA and TfR was 2 mg/mL, 0.1%, and 1 – 60 μg/mL respectively. The particles were incubated with receptor for 1 hour at room temperature before analysis by differential centrifugal sedimentation (DCS).
DCS measurements were performed as discussed above. Samples which had not been incubated with TfR yet had undergone the same treatment i.e. the same BSA conc. and the same incubation time, were injected as a reference. To make sure the initial measurement was correct, the particles without any receptor were measured three times and the average size was taken

***In Vitro* characterization of particle functionality**

Cell culture

Tissue culture reagents were purchased form GIBCO Invitrogen Coporation/ Life Technologies Life Sciences unless otherwise specified. The adherent tumour cell line A549 (ATCC- CCL-185) were maintained in monolayer cultures in MEM supplemented with 10% fetal bovine serum. Cells were cultured in an incubator at 37 °C with 5% CO_2/95% air and saturated humidity. Cell line was confirmed to be mycoplasma negative using the MycoAlert™ Kit (Lonza) and were tested monthly.

Cell silencing and flow cytometry

A total of 13,000 cells were seeded in 24 well plates and incubated for 24 hours prior to silencing of the gene coding for transferrin receptor (TFRC). Cells were transfected with 15 pmol of Silencer Select siRNA siTFRC using Oligofectamine™ according to the manufacturer's instructions. Neg1 silencer was used as a negative control. Cells were transfected with siRNA for 72 hours prior to exposure to nanoparticles or labelled transferrin.
To exposure the cells to nanoparticles, after 72 hours silencing, cells were washed for 20 minutes in serum-free MEM. The medium was aspirated and nanoparticle suspensions were applied to the cells. Nanoparticle suspensions were freshly prepared by diluting the nanoparticle stock solution in serum-free MEM. Similar experiments were performed by exposing cells to a solution of 5 μg/ mL Alexa488 labelled human transferrin in serum-free MEM.
For flow cytometry, the cells were washed once in MEM supplemented with 10 % FBS (v/v), twice with PBS and harvested with trypsin. Cell pellets were fixed at room temperature with 4% formalin (Sigma-Aldrich) in PBS for 20 minutes and resuspended in PBS. Cell-associated fluorescence (15,000 cell per sample) was detected using an Accuri C6 (BD Biosciences). The results are reported at the median of the distribution of cell fluorescence (excitation: 488 nm, filter: 530/30 nm), averaged over 2-3 independent replicates. Error bars represent 1 standard deviation of the mean between replicates.
The receptor specific fraction is determined by the difference in median cell fluorescence intensity (following 6 hours exposure) between the non-silenced control and silenced cells, divided by the non-silenced control intensity as reflected in Eq 4.

NMR Spectra

APTS

^{1}H NMR (400 O+], ˉHXWHULXP 2[LGH) į 2.41 (W, *J* = 6.9 Hz, 1H), 1.42 – 1.22 (m, 1H), 0.37 – 0.18 (m, 1H).

SM(PEG)$_8$

^{1}+ 105 (400 O+], 'HXWHULXP 2[LGH) į 6.38 – 5.62 (m, 1H), 3.70 – 3.12 (m, 25H), 2.36 – 2.20 (m, 3H), 1.04 (t, *J* = 7.1 Hz, 0H).

APTS on particles

^{1}+ 105 (600 O+], 'HXWHULXP 2[LGH) į 2.99 – 2.81 (m, 1H), 1.64 (d, *J* = 8.6 Hz, 1H), 0.63 – 0.49 (m, 1H).

SM(PEG)$_8$ on particles

^{1}H NMR (400 MHz, Deuterium Oxide) į 3.57.

APTS after dissolution

^{1}+ 105 (400 O+], 'HXWHULXP 2[LGH) į 2.43 (W, *J* = 7.0 Hz, 1H), 1.48 – 1.28 (m, 1H), 0.44 – 0.16 (m, 1H).

SM(PEG)$_8$ after dissolution

^{1}+ 105 (400 O+], 'HXWHULXP 2[LGH) į 6.26 – 5.67 (m, 0H), 3.77 – 3.41 (m, 1H), 3.41 – 2.96 (m, 0H), 2.84 (s, 0H), 2.58 (s, 0H).

Calculations

Assumptions were made in all calculations and applied across all the work presented:

- All particles are perfect spheres of the same size.
- The density of all particles is the same and equal to 2 g/cm^3.
- DCS Wt. Mean gives an accurate description of "true size"

Number of Nanoparticles (Eq 1)

$$[illegible] = \frac{3.[illegible].10^{[illegible]}}{[illegible].4.[illegible].[illegible]^3}$$

[illegible] = [illegible] (measured through vaccum drying of aliquots of known volume)

[illegible] = [illegible] (taken as 2.0 g/cm^3)

[illegible] = [illegible] (taken from DCS analysis)

Total Surface Area (Eq 2)

$$[illegible] = [illegible].4[illegible]^2$$

TGA determination of surface PEG density (Eq 3)

$$[illegible] = \frac{[illegible].[illegible]}{[illegible].[illegible]}$$

[illegible] = [illegible]

[illegible] = [illegible]

[illegible] = [illegible]

[illegible] = [illegible] (radius taken from DCS analysis of scaffold NP)

[illegible] = [illegible]

Receptor specific uptake fraction (Eq 4)

[illegible]

= [illegible]

[illegible] = [illegible]

[illegible]

[illegible] = [illegible]

[illegible]
